# Conservative production of galactosaminogalactan in *Metarhizium* is responsible for appressorium mucilage production and topical infection of insect hosts

**DOI:** 10.1371/journal.ppat.1009656

**Published:** 2021-06-14

**Authors:** Lijuan Mei, Xuewen Wang, Ying Yin, Guirong Tang, Chengshu Wang

**Affiliations:** 1 CAS Key Laboratory of Insect Developmental and Evolutionary Biology, CAS Center for Excellence in Molecular Plant Sciences, Shanghai Institute of Plant Physiology and Ecology, Chinese Academy of Sciences, Shanghai, China; 2 CAS Center for Excellence in Biotic Interactions, University of Chinese Academy of Sciences, Beijing, China; 3 School of Life Science and Technology, ShanghaiTech University, Shanghai, China; McGill University, CANADA

## Abstract

The exopolysaccharide galactosaminogalactan (GAG) has been well characterized in Aspergilli, especially the human pathogen *Aspergillus fumigatus*. It has been found that a five-gene cluster is responsible for GAG biosynthesis in Aspergilli to mediate fungal adherence, biofilm formation, immunosuppression or induction of host immune defences. Herein, we report the presence of the conserved GAG biosynthetic gene cluster in the insect pathogenic fungus *Metarhizium robertsii* to mediate either similar or unique biological functions. Deletion of the gene cluster disabled fungal ability to produce GAG on germ tubes, mycelia and appressoria. Relative to the wild type strain, null mutant was impaired in topical infection but not injection of insect hosts. We found that GAG production by *Metarhizium* is partially acetylated and could mediate fungal adherence to hydrophobic insect cuticles, biofilm formation, and penetration of insect cuticles. In particular, it was first confirmed that this exopolymer is responsible for the formation of appressorium mucilage, the essential extracellular matrix formed along with the infection structure differentiation to mediate cell attachment and expression of cuticle degrading enzymes. In contrast to its production during *A*. *fumigatus* invasive growth, GAG is not produced on the *Metarhizium* cells harvested from insect hemocoels; however, the polymer can glue germ tubes into aggregates to form mycelium pellets in liquid culture. The results of this study unravel the biosynthesis and unique function of GAG in a fungal system apart from the aspergilli species.

## Author summary

Fungal cell wall associated exopolymer GAG was first identified from *Aspergillus niger* decades ago and has been well characterized in terms of its biosynthetic mechanism, pathological and biological functions in *Aspergillus* fungi, especially the human pathogen *A*. *fumigatus*. With the accomplishment of diverse fungal genomes, the conserved GAG biosynthetic gene cluster is found to patchily distribute in fungal species beyond Aspergilli. However, until this study, the contribution of the clustered genes to GAG production has not been investigated in any non-*Aspergillus* fungi. We performed gene deletions and functional characterizations of GAG in an insect pathogenic fungus *Metarhizium robertsii*, and found that the partially acetylated GAG can be produced by the fungus to contribute to fungal virulence during topical infection of insect hosts. This exopolymer can be produced on germ tubes, mycelia and appressoria to mediate cell adherence, cuticle penetration, biofilm formation, mycelium aggregation in liquid culture and in particular, appressorium mucilaginous matrix formation. In addition, it was found that loss of GAG production could alter appressorium protein expressions including the enzymes involved in degrading insect cuticles. Our data advance the understanding of GAG biosynthesis and functions in an animal pathogenic fungus that is highly divergent from Aspergilli.

## Introduction

Cell wall components and integrity play essential roles in maintaining fungal physiology and stress responses as well as the infection biology of the animal and plant pathogens [1,2]. The cell wall constituents of the β-glucans, chitin and galactomannan of different pathogenic fungi have long been recognized as the pathogen-associated molecular patterns (PAMPs) to induce host immune responses in both plants and animals [1,3,4]. Fungi can also produce different exopolysaccharides (EPSs) with diverse biological activities in association with fungal cell walls [5]. The exopolymer galactosaminogalactan (GAG) was first identified from the filamentous fungus *Aspergillus niger* and has so far only been functionally characterized in *Aspergillus* species [6,7]. Polymer GAG is biosynthesized by *Aspergillus* on germ tubes and mycelia but not on the resting conidia [8], which is structurally composed of α-1,4 linked galactose (Gal), N-acetyl galactosamine (GalNAc) and galactosamine (GalN) [9]. This polysaccharide has been demonstrated as an important virulence factor of *A*. *fumigatus* by mediating the adherence of hyphae to host cells [10–12], immunosuppression [8,13], and as a PAMP to elicit host immune responses [14,15]. Similar to the bacterium EPS Pel [16], GAG can also mediate biofilm formation to increase the resistance of *A*. *fumigatus* against antifungal drugs [17,18]. In addition, it has been found that the cell wall GAG as well as α-1,3-glucan can mediate the aggregation of *A*. *oryzae* hyphae to form mycelium pellets in liquid culture [19,20]. Production of the EPSs containing α-1,4 linked GalNAc and GalN has also been reported in a few non-*Aspergillus* ascomycete fungi such as *Neurospora crassa*, *Penicillium frequentans* and *Paecilomyces* sp. [21–23], and the basidiomycete *Trichosporon asahii* [11]. Until this study, biological effects of GAG have not been reported in divergent fungal species apart from Aspergilli.

By using the mutants of *A*. *fumigatus* with adhesive defects in combination of comparative transcriptomics analysis, an epimerase gene termed *Uge3* was first identified with contribution to GAG production [10]. It was later found that a co-regulated five-gene cluster including *Uge3* and its close neighbors spherulin *Sph3*, alpha-1,4-galactosaminidase *Ega3*, glycosyltransferase *Gtb3* and deacetylase-like gene *Agd3* is jointly required for GAG biosynthesis and modification in *A*. *fumigatus* [11,24,25]. Enzyme activity and structure analyses suggested that GAG biosynthesis starts with the conversion of UDP-glucose and UDP-N-acetyl glucosamine into UDP-galactose and UDP-GalNAc by Uge3, and the products will then be putatively linked and exported by Gtb3 [26]. A recent study has shown that Gtb3 (also called Gt4c) is required for GalN production and therefore GAG biosynthesis [15]. The GalNAc moieties within the newly biosynthesize polymer can be deacetylated to GalN by the deacetylase Agd3, which belongs to the carbohydrate esterase family CE18 and contains an unique carbohydrate binding module (CBM87) to aid in GAG deacetylation by binding to its GalN-GalNAc rich region [11,12]. Sph3 has a glycoside hydrolase (GH) activity and was suggested to function for GAG cleavage and maturation [24]. Ega3 contains a GH114 (for GH family 114) domain that can disrupt GAG-dependent biofilms by targeting GalN region of GAG [25]. This heteropolysaccharide has been successfully synthesized by optimized chemical reactions [9]. Biochemically, however, GAG chain elongation, exportation and modification remain elusive. Genome survey indicated that the putative GAG biosynthetic gene cluster is present in 28 out of 250 fungal species with publically available genome information. All these cluster-containing fungi largely belong to the ascomycete Pezizomycotina subphylum plus a basidiomycete species *Trichosporon asahii* [11]. However, GAG biosynthesis and biological functions remain unclear in these non-*Aspergillus* fungi.

Ascomycete insect pathogenic fungi such as *Metarhizium robertsii* have been developed as insect biocontrol agents and investigated as genetically tractable system for fungus-insect interactions [27,28]. *Metarhizium* infection starts from spore adhesion to insect cuticles and the spores then germinate to produce the infection structure appressoria for penetration of insect cuticles. After reaching insect hemocoels, fungal cells will switch from filamentous growth to yeast-type budding to quickly occupy insect body cavity and kill the hosts [29]. It has been found that two divergent adhesins secreted by *Metarhizium* are required for mediating spore adhesion either to insect cuticles (Mad1) or to plant surfaces (Mad2) [30]. In combination with the buildup of the appressorium turgor pressure by degradation of cellular lipid droplets [31,32], the characteristic mucilaginous matrix will be produced around appressoria to assist the secretion and function of degradation enzymes such as serine proteases and chitinases [33–35]. Once entering insect body cavity, fungal cell wall structures will be remodified to reduce the PAMP contents of glucans and chitins along with the secretion of the coat proteins to evade insect immunities [36–38]. It is still unclear whether additional factor(s) involved in mediating spore adhesion and how the appressorium mucilage is produced by *Metarhizium* fungi.

In this study, we found that the putative GAG biosynthetic gene cluster is conservedly present in the genomes of *Metarhizium* species. By gene deletions in *M*. *robertsii*, it was verified that the conserved cluster is responsible for GAG production in this insect pathogen to mediate appressorium mucilage production, cuticle penetration and fungal topical infection of insect hosts.

## Results

### Stage-specific biosynthesis of GAG by *M*. *robertsii*

**Fig 1:**
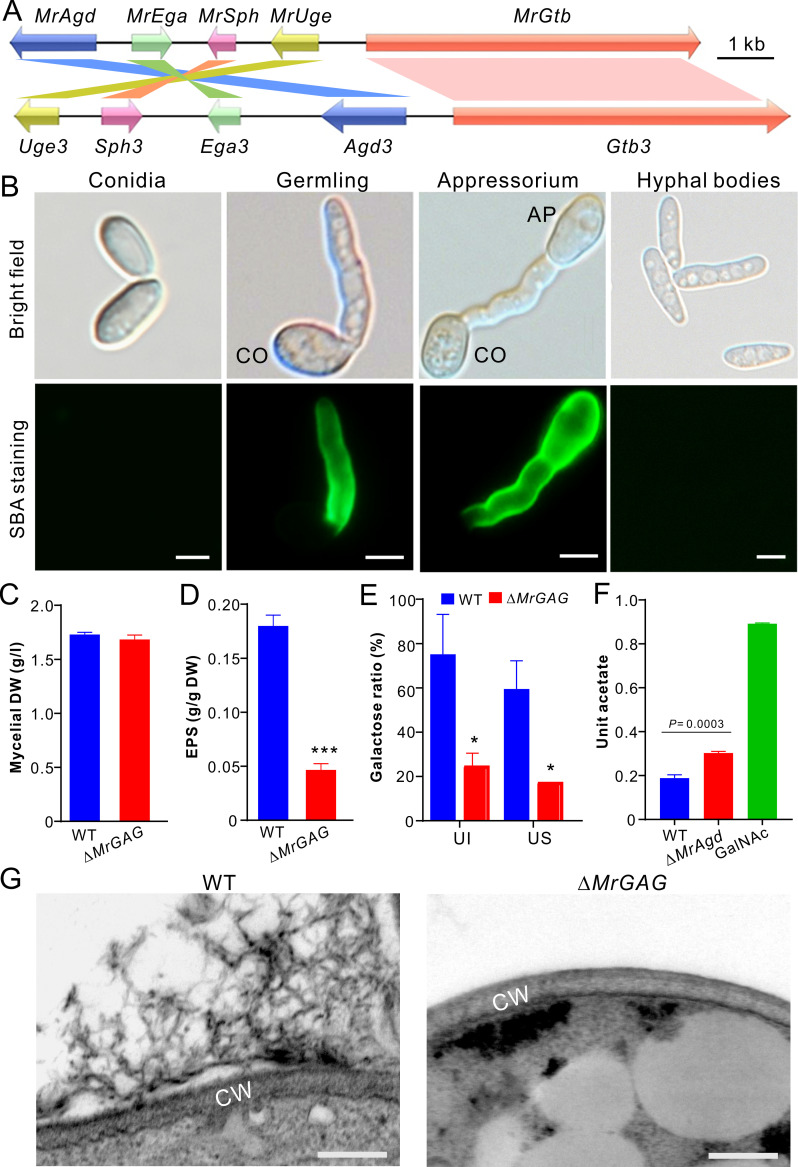
Biosynthesis of GAG in *M*. *robertsii*. (A) Mesosyntenic relationship of the GAG biosynthetic gene clusters between *M*. *robertsii* and *A*. *fumigatus*. (B) Variation of GAG production by different type of cells. CO, conidium; AP, appressorium. Bar, 5 μm. (C) No difference of mycelium biomass production between WT and Δ*MrGAG*. The fungi were grown in SDB for three days and the mycelia were harvested, dried and weighted. DW, dry weight. (D) Comparison of the crude exopolysaccharide (EPS) production between WT and Δ*MrGAG*. Fungal samples were collected three days post inoculation in SDB. The urea-insoluble pellets were freeze-dried and compared. The significance of the two-tailed Student’s *t*-test difference is at: ***, *P* < 0.001. (E) Comparison of the galactose content hydrolyzed from the EPS produced by WT and Δ*MrGAG*. Fungal samples were harvested three days post inoculation in SDB. UI, urea insoluble EPS sample; US, urea soluble EPS sample. (F) Comparative quantification analysis of GAG acetylation. The EPS harvested from the WT and Δ*MrAgd* culture filtrates were hydrolyzed prior to the analysis of both reducing sugar and acetate. Unit acetate is referred to the acetate content out of the reducing sugar within each hydrolyzed sample. The standard GalNAc was hydrolyzed and used as a control. (G) Mycelial surface structure differences. The WT and Δ*MrGAG* mycelia were harvested from the day 3 SDB cultures for TEM analysis. Bar, 100 nm. CW, cell wall.

By genome survey, we found that the conserved GAG biosynthetic gene cluster (termed *MrGAG*) is present in *M*. *robertsii* including five genes (termed *MrAgd*, *MrEga*, *MrSph*, *MrUge* and *MrGtb*, respectively), which demonstrated a mesosyntenic relationship to that of *A*. *fumigatus* with gene order and orientation differences (Fig 1A). Besides Aspergilli, this gene cluster is also patchily but conservedly distributed in the genomes of different *Metarhizium* species and other divergent fungal species including the plant pathogenic fungi such as *Verticillium dahliae* and *Botrytis cinerea*, the nematode trapping fungus *Arthrobotrys oligospora* and the model fungus *Neurospora crassa* (S1 Table). The identities at the amino acid level vary from 28% (the putative glycosyltransferase MrGtb versus Gtb3) to 51% (the putative UDP-glucose-4-epimerases MrUge versus Uge3) between the orthologous proteins of *M*. *robertsii* and *A*. *fumigatus* (S1 Table). To verify the production of GAG-like polymer by *M*. *robertsii*, we prepared different type of cells for staining with the GalNAc specific and fluorescein-conjugated lectin soybean agglutinin (SBA) [10]. The results indicated that the fluorescent signal could be detected on the germ tubes and appressorium cells induced on a hydrophobic surface but not on conidial spores and the hyphal bodies harvested from the infected insect hemolymph (Fig 1B). Thus, consistent with the finding in *A*. *fumigatus* [8], GAG can be produced by *M*. *robertsii* in a developmental-stage dependent manner.

To further verify the GAG production by *M*. *robertsii*, we deleted the complete gene cluster and individual genes by homologous replacements. The obtained mutants were verified by PCR and reverse transcription (RT)-PCR (Figs S1A and 1B). After the growth in the Sabouraud dextrose broth (SDB), we found that the clustered genes could all be transcribed by the wild type (WT) strain, and deletion of one gene did not alter the expression level of other ones when compared with the transcribed abundances by the WT (S1B Fig). Gene deletions had no obvious negative effects on fungal growth and sporulation on potato dextrose agar (PDA) (S2A Fig). In addition, both WT and Δ*MrGAG* grew similarly on a minimum medium or PDA amended with different components for stress challenges (S2B Fig). After the growth in SDB for three days, the mycelial biomasses had no difference between WT and Δ*MrGAG* (Fig 1C). We then extracted the extracellular polysaccharides after fungal growth in SDB. Quantification analysis revealed that both the exopolysaccharide GAG and its specific monosaccharide galactose were significantly reduced in Δ*MrGAG* when compared with those of the WT strain (Fig 1D and 1E). Acidic hydrolysis and acetate quantification analysis revealed a significant (*P =* 0.0003) difference between the WT and null mutant of the deacetylase gene *MrAgd* (Fig 1F). Relative to the GAG produced by the WT strain, the deacetylation degree of the GAG mediated by MrAgd reached 37.54% ± 4.37. Further mass spectrometry analysis detected the monosaccharides galactose and GalN from the hydrolytic EPS samples of WT and Δ*MrAgd*. Similar to the acidic hydrolysis of GalNAc, GalNAc constituent within the EPS samples was hydrolyzed to GalN (S3 Fig). We also found that, in contrast to the WT, the GAG-associated extracellular fibrils disappeared outside the cell walls of Δ*MrGAG* after transmission electron microscopy (TEM) analysis (Fig 1G). Thus, it is confirmed that this conserved gene cluster is responsible for GAG production in *M*. *robertsii*.

### GAG production mediates the formation of appressorial mucilage and mycelial pellets

**Fig 2:**
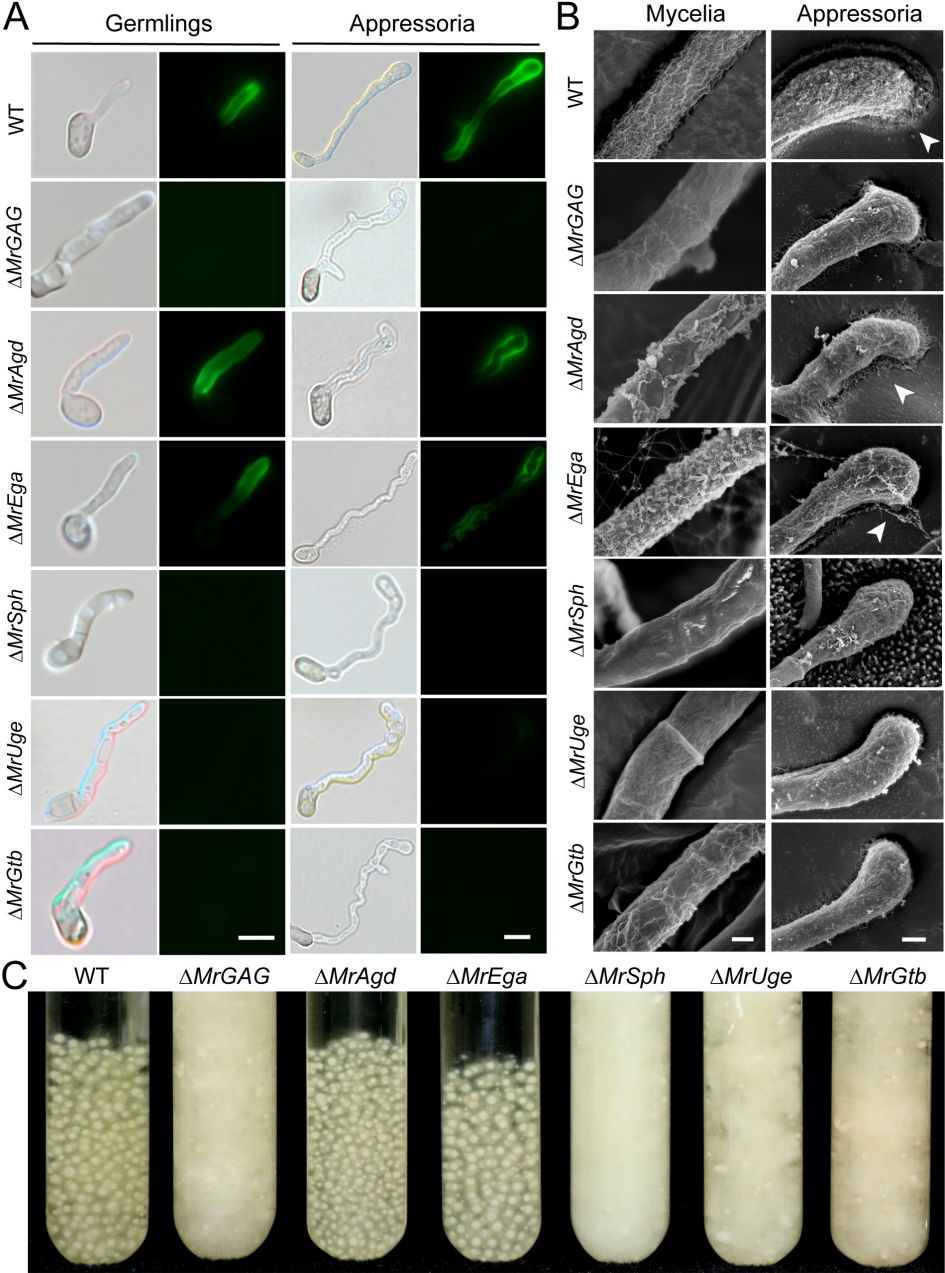
Microscopic and liquid-culture phenotyping. (A) Fluorescent staining of the WT and mutant cells. Fungal cells were germinated in SDB for 12 hrs or induced for appressorium production in a MM medium for 18 hrs prior to the staining with SBA. Bar, 10 μm. (B) SEM observation of fungal mycelium and appressorium cells. The mycelia of the WT and mutants harvested from the day 3 SDB medium were used for SEM analysis. The appressoria of the WT and mutants were induced on the mealworm front wings. Mucilage matrixes produced by the WT, Δ*MrAgd* and Δ*MrEga* appressoria are arrowed. Bar, 200 nm. (C) Phenotyping of the liquid cultures of the WT and different mutants. Relative to the WT, production or non-production of mycelial pellets was observed for different mutants after the growth in SDB for three days.

We found that deletion of the whole cluster or individual gene did not impair the formation of the infection structure appressoria. After the germination in SDB and induction of appressorium formations of the WT and mutants, SBA staining indicated that the fluorescent signal could not be detected on the surface of both the germ tube and appressorium cells of Δ*MrSph*, Δ*MrUge* and Δ*MrGtb* but on the cells of Δ*MrAgd* and Δ*MrEga* (Fig 2A). The mutants Δ*MrAgd*, Δ*MrSph* and Δ*MrUge* were complemented, and positive signals could be detected on the surface of these rescued strain cells (S4A Fig). Consistently, the scanning electron microscopy analysis (SEM) revealed that the surface decorations associated with GAG disappeared on the hyphal cells of Δ*MrSph*, Δ*MrUge* and Δ*MrGtb* but more or less on the cells of Δ*MrAgd* and Δ*MrEga* (Fig 2B). For the appressorial cells induced on the insect wings, SEM analysis demonstrated the formation of the mucilaginous matrix around the WT cells but not on Δ*MrGAG*. For individual mutants, a reduced level of appressorial mucilage could also be observed around Δ*MrAgd* and Δ*MrEga* cells but not on other null mutants when compared with that of the WT (Fig 2B).

**Fig 3:**
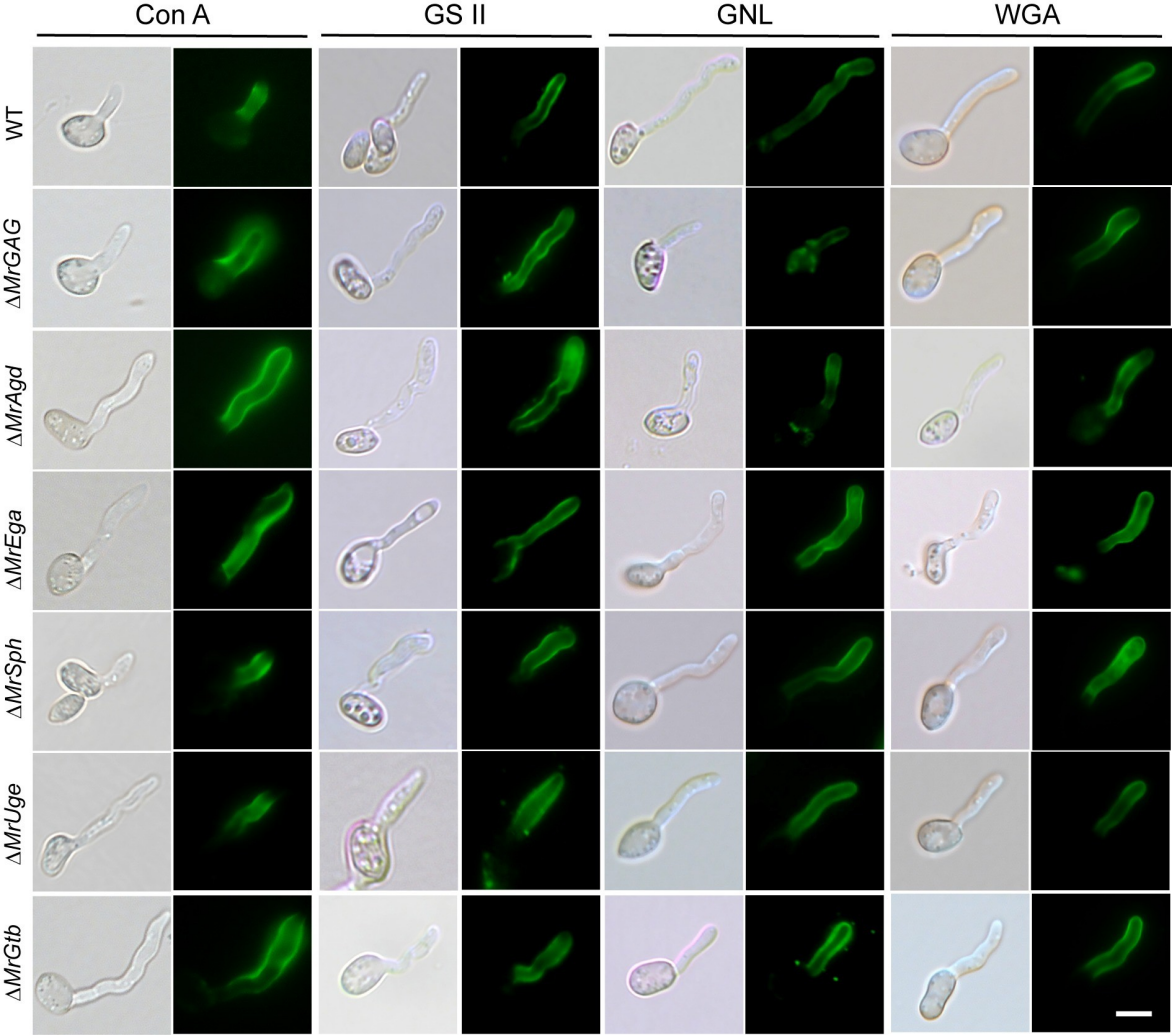
Lectin staining of the WT and mutant germlings for different cell wall constituents. The WT and mutant spores were germinated in SDB and then stained with different fluorescent lectins: ConA, Concanavalin A for detecting α-mannopyranosyl and α-glucopyranosyl residues; GSII for detecting α- or β-linked *N*-acetyl-*D*-glucosamine); GNL, *Galanthus nivalis* lectin for detecting α-1,3-mannose; WGA, wheat germ agglutinin for selective binding *N*-acetyl-*D*-glucosamine and *N*-acetylneuraminic acid residues.

We also examined whether the impairment of GAG production would affect cell wall constituents by fluorescent staining with different fluorescein-labeled lectins including Concanavalin A (Con A), GSII, GNL and wheat germ agglutinin (WGA). As a result, no obvious differences of the fluorescent signals were found on germ tubes between WT and mutants (Fig 3). For example, the fluorescent signals of Con A (staining α-mannopyranosyl and α-glucopyranosyl residues) and WGA (for selective binding *N*-acetyl-*D*-glucosamine and *N*-acetylneuraminic acid residues) were similarly detected on the WT and different mutant cells (Fig 3). Thus, disruption of the GAG production might not interfere the overall constituent of the *Metarhizium* cell walls. Nevertheless, additional phenotype difference was observed between WT and mutants. It was found that the SBA-staining positive strains (i.e., WT, Δ*MrAgd* and Δ*MrEga*) could form mycelial pellets in liquid cultures whereas the GAG non-producing mutants (i.e., Δ*MrGAG*, Δ*MrSph*, Δ*MrUge* and Δ*MrGtb*) produced flocculent mycelia without pellet formation (Fig 2C). Gene complementations restored the ability of Δ*MrSph* and Δ*MrUge* to produce mycelial pellets while Δ*MrAgd*-CP produced the pellets like the WT and Δ*MrAgd* (S4B Fig). A time-course monitoring of the WT and Δ*MrGAG* spore germinations in liquid medium revealed that GAG production could promote the WT cells to aggregate nine hours post inoculation and form sizeable pellets within 12 hours (S5 Fig).

### GAG biosynthesis is required for fungal topical infection of insects

**Fig 4:**
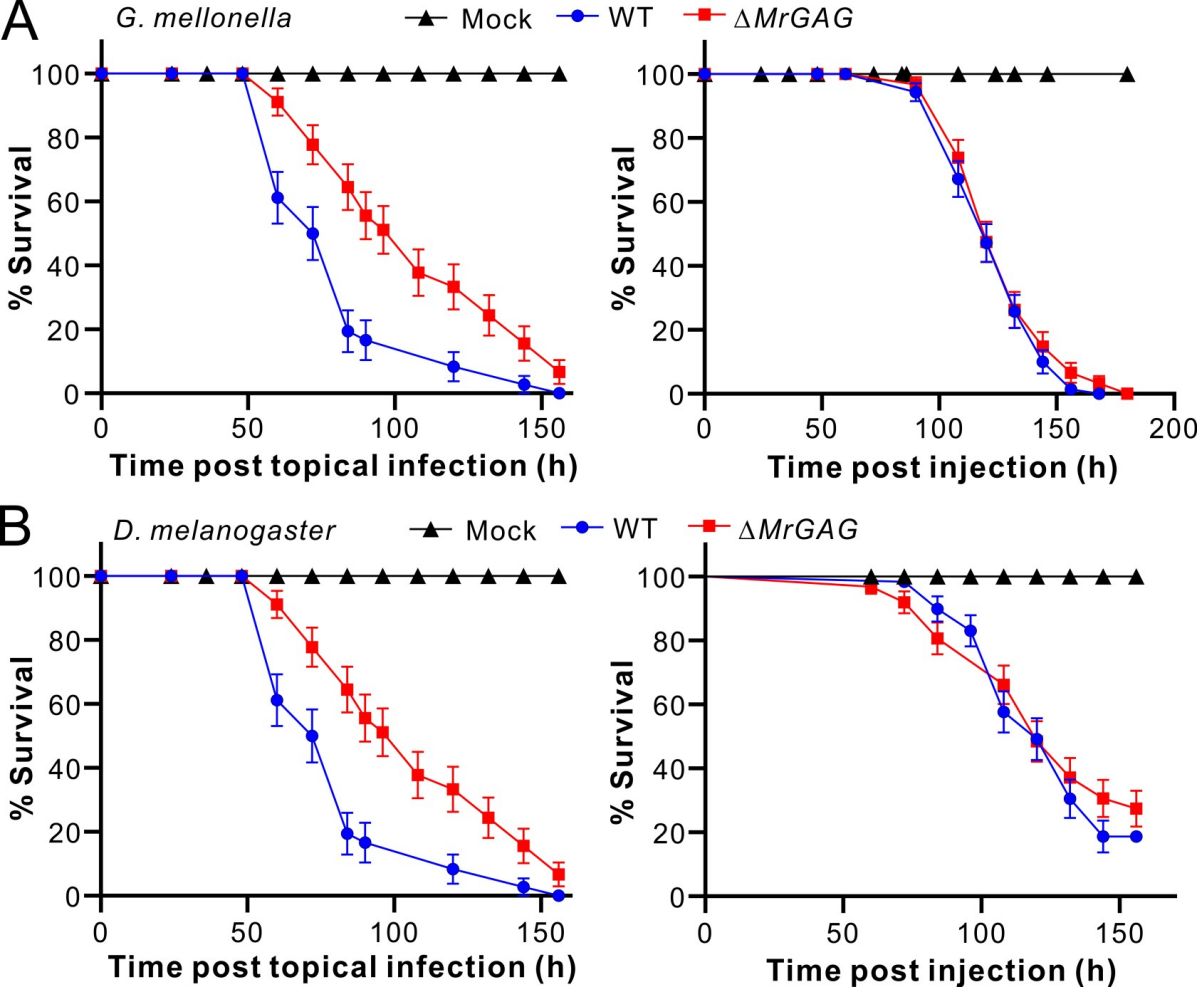
Insect survivals after infections by WT and Δ*MrGAG*. (A) Topical infection (left panel) and injection (right panel) assays against the last instar larvae of wax moth *G*. *mellonella*. (B) Topical infection (left panel) and injection (right panel) assays against the female adults of the fruit fly *D*. *melanogaster*. Insects treated with 0.05% Tween-20 were used as a mock control.

GAG has been verified to be a virulence factor of *A*. *fumigatus* with multiple contributions [6,13,14]. To determine whether GAG production is also required for *M*. *robertsii* infection of insect hosts, we performed both topical infection and injection assays with the WT and Δ*MrGAG* spores against the wax moth (*Galleria mellonella*) larvae and the female adults of the fruit fly *Drosophila melanogaster*. The result indicated that, relative to the WT treatment, the survivals of the wax moth larvae were significantly (Log-rank test: χ^2^ = 7.62; *P* = 0.006) delayed during topical infection by Δ*MrGAG*. However, no obvious difference (χ^2^ = 0.89; *P* = 0.35) of insect survivals was observed between WT and mutant during injection assays (Fig 4A). Likewise, substantial difference was evident between WT and Δ*MrGAG* during topical infection (χ^2^ = 11.64; *P* = 0.0006) but not during injection (χ^2^ = 0.62; *P* = 0.43) of the fruit flies (Fig 4B). It was also found that, relative to the WT, deletion of the individual gene could led to the significant (*P* < 0.05) reduction of the virulence during topical infection of the wax moth larvae, including the SBA-staining positive mutants Δ*MrAgd* and Δ*MrEga* (S2 Table). Thus, GAG production is required for the full virulence of *M*. *robertsii* during topical infection of insect hosts.

### GAG mediates surface adhesion, biofilm formation and cuticle penetration

**Fig 5:**
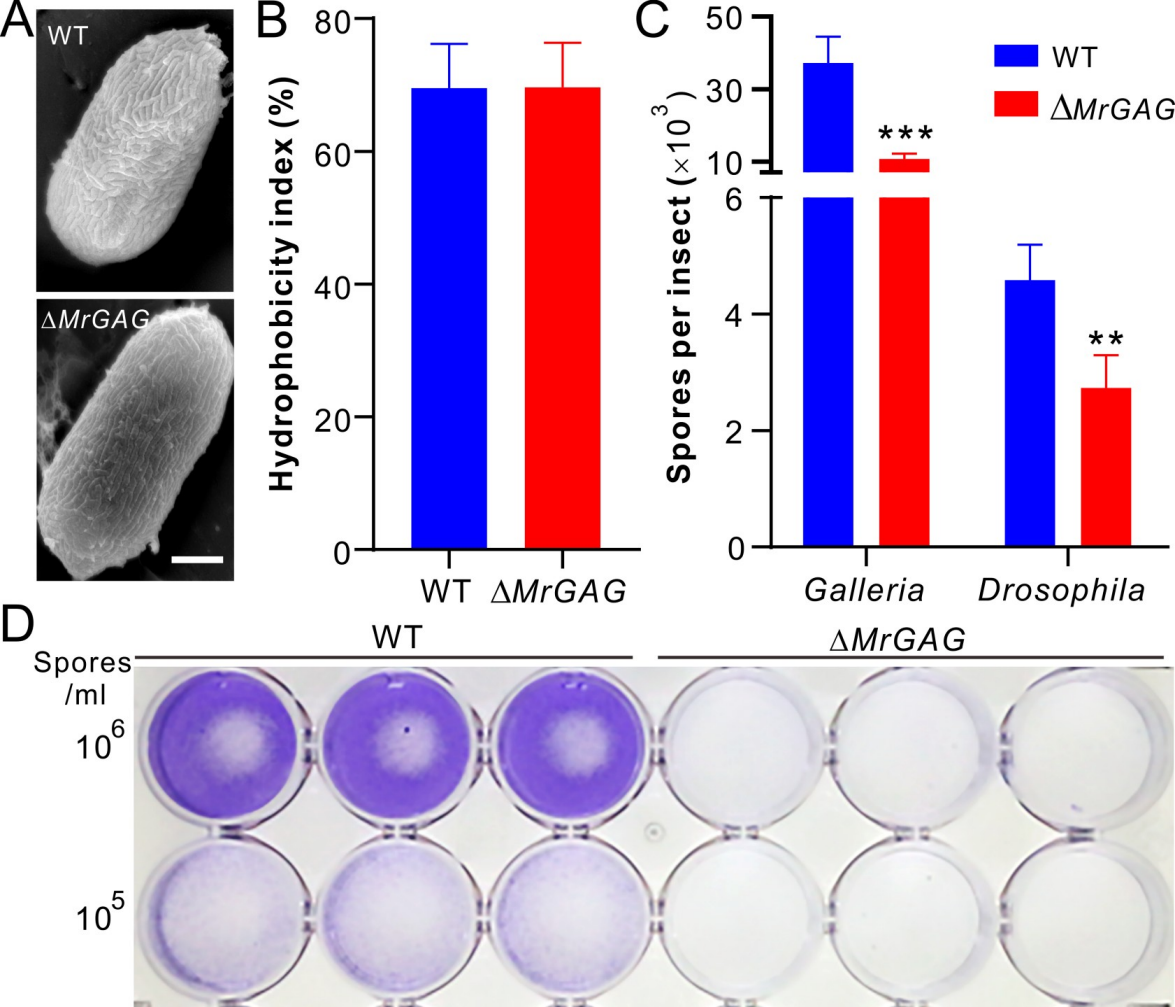
Conidial hydrophobicity and adhesion assays. (A) SEM observation of the WT and mutant conidia. Bar, 2 μm. (B) No difference of the conidial spore hydrophobicity between WT and Δ*MrGAG*. (C) Variation of the spore adhesion abilities between WT and Δ*MrGAG* toward different insects. The spores were washed off and counted eight hours post treatment. The significance of the two-tailed Student’s *t*-test difference is at: ***, *P* < 0.001; **, *P* < 0.01. (D) Variation of the spore adhesion ability between WT and Δ*MrGAG* toward hydrophobic surface. Different concentration of spore suspensions were inoculated into the 24-well plate for 24 hrs and then washed off with PBS buffer. The wells were stained with crystal violet before imaging.

Having shown that GAG is required for fungal pathogenesis during topical infection, we then performed experiments to determine the pathogenesis-related effect of this EPS. SEM analysis revealed that the hydrophobin rodlets on conidial surface were not impaired after deletion of the gene cluster when compared with that of the WT (Fig 5A). Consistently, spore hydrophobicity tests revealed no difference between WT and Δ*MrGAG* (Fig 5B). Similar conidial surface structures were also observed between WT and individual mutants (S6A Fig), thus the additional supports of the non-production of GAG on conidial spores. We then examined spore adherence towards both the insect cuticles and hydrophobic surfaces after inoculation of the WT and Δ*MrGAG* conidia for different times. It was found that the amounts of the Δ*MrGAG* spores/germlings adhered to both the wax moth larvae and fruit fly adult cuticles were significantly lower (*P* < 0.01) than those on the WT-treated insects eight hours post immersion with the spore suspensions (Fig 5C). Twenty-four hours post inoculation on hydrophobic surface, it was found that the biofilms formed dosage-dependently by the WT cells could be hardly washed off, which were in sharp contrast to the inoculations of the Δ*MrGAG* spores (Fig 5D). The test of individual gene deletion and complementation mutants indicated that deletion of any single gene impaired fungal ability to form biofilms while gene rescues restored mutant defects (S4C Fig).

**Fig 6:**
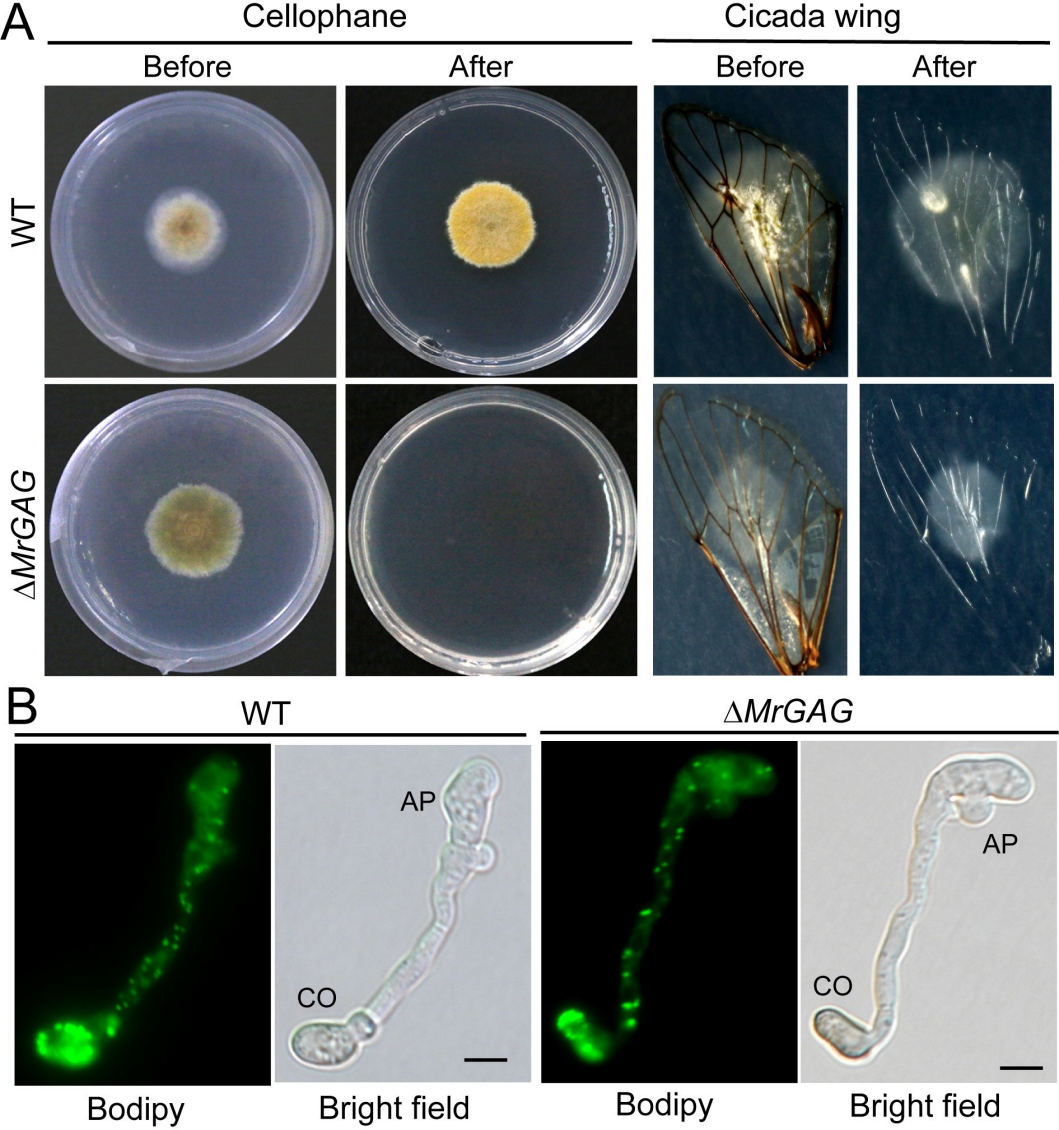
Penetration and appressorium formation assays. (A) Fungal penetration assays. Both the WT and Δ*MrGAG* were inoculated on cellophane membrane for three days or cicada wings for 40 hrs. The membrane and insect wings were then carefully removed with fungal cultures, and the plates were kept for incubation for another one week before photographing. (B) Appressorium formation and lipid-droplet utilization by WT and Δ*MrGAG*. Appressorium were induced on hydrophobic surfaces for 18 hrs and stained with the fluorescent dye Bodipy. CO, conidium; AP, appressorium. Bar, 5 μm.

We also examined the penetration abilities of the WT and mutant strains. The results indicated that deletion of the GAG biosynthetic gene cluster impaired fungal ability to penetrate both the cellophane membrane and cicada wings when compared with the WT (Fig 6A). The similar defects were found for the individual gene-deletion mutants (S6B and S6C Fig). In support to the finding of the Δ*MrGAG* penetration defect, it was found that the number of the mutant propagules (i.e. hyphal bodies) formed in insect hemolymph was significantly lower than that of the WT after the same time of topical infection (S7 Fig). On the other hand, we found there was no obvious difference between WT and Δ*MrGAG* in terms of the mobilization of lipid droplets from the conidial cells into appressoria for the degradation and generation of turgor pressure (Fig 6B).

### Association of GAG production with protein expressions in appressoria

Considering that GAG was highly produced during appressorium development for mucilage formation, we performed comparative proteomic analysis using the WT and Δ*MrGAG* appressorial samples induced on cicada wings for 24 hours (S8A Fig). Differential expression of hundreds of proteins was evident between WT and Δ*MrGAG* (S8B Fig and S3 Table). Gene enrichment analysis indicated that a higher number of proteins associated with cell wall component was downregulated in Δ*MrGAG* when referred to that of the WT (S8C and S8D Fig). It was noted that, among the five GAG-biosynthetic enzymes, two of them (i.e., MrAgd and MrEga) were detected in the WT samples. To examine the reliability of proteomic analysis, we performed a parallel reaction monitoring (PRM) analysis [39]. As a result, 15 of the 17 examined proteins had similar up- or down-accumulation patterns between the PRM and proteomic analysis (S4 Table). In particular, the peptide fragment of MrAgd was detected from the WT sample but absent from that of Δ*MrGAG* (S4 Table).

Particular interests were then paid to examine those enzymes involved in degradation of insect cuticles such as peptidases and glycoside hydrolases (GHs) as well as those putatively involved in stress responses (S9 Fig). For example, relative to the WT, 22 putative peptidases were differentially expressed in the mutant appressoria (S3 Table). Likewise, 11 GH enzymes were down-regulated whereas 10 up-regulated in the mutant when compared with those of the WT strain. Relative to the WT, a few lipases such as an extracellular lipase (MAA_08386; 1.32-fold) were down-regulated in Δ*MrGAG* (S3 Table). Otherwise, deletion of the GAG biosynthetic gene cluster could trigger the up-regulation of more proteins such as the heat shock proteins and putative glutathione S-transferases involved in stress responses when compared with the WT during appressorium formation (S9 Fig and S3 Table). Consistent with the aforementioned reduction of the mutant adhesion ability (Fig 4C), the adhesin protein Mad1 (MAA_03775), mediating fungal adhesion to host cuticles [30], was detected with a reduced level of 1.84-fold in Δ*MrGAG* when compared with that of the WT (S3 Table).

### Effects of GAG production on hydrolytic enzyme activities

**Fig 7:**
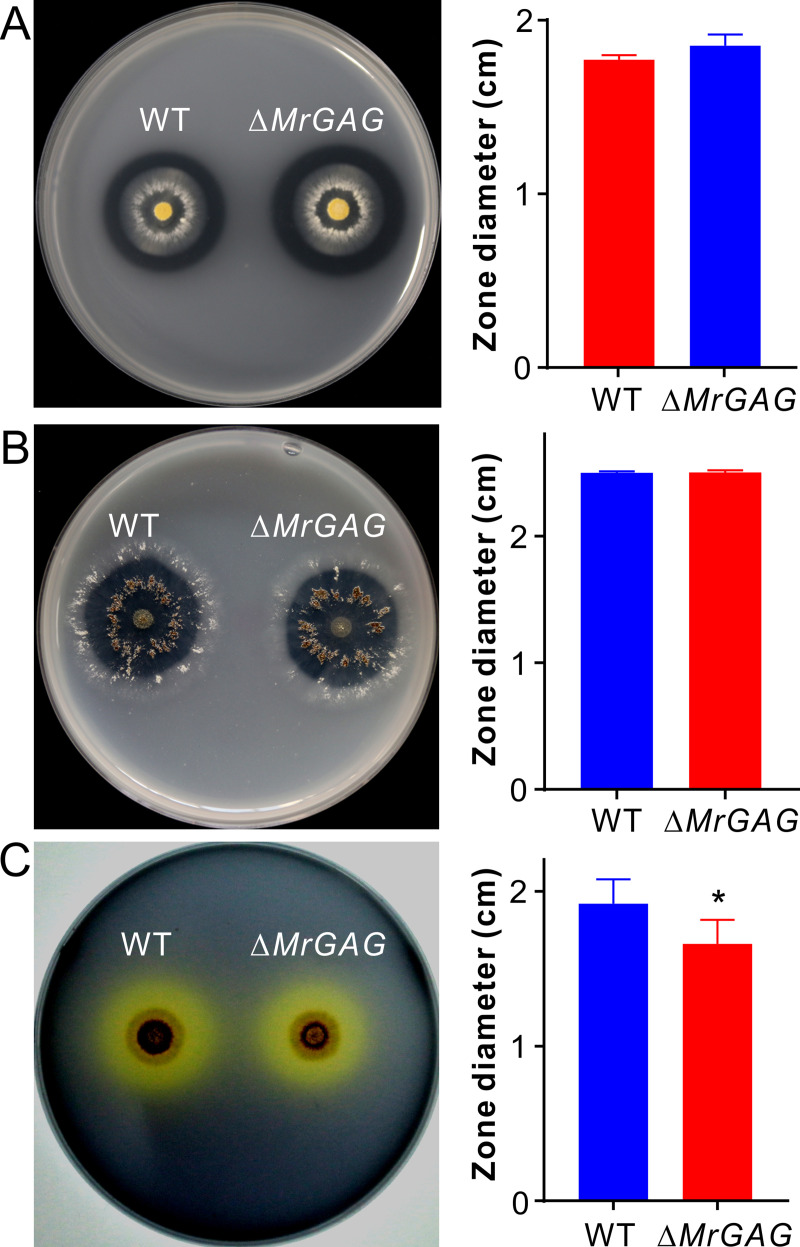
Comparative enzyme hydrolytic activity assays between WT and Δ*MrGAG*. (A) Non-significant variation between strains in digestion of casein seven days post inoculation. (B) Non-significant variation between strains in digestion of chitin seven days post inoculation. (C) Variation between strains in digestion of olive oil five days post inoculation. The right part of each panel shows the data of the hydrolytic zone diameters formed by WT and Δ*MrGAG*. The significance of the two-tailed Student’s *t*-test difference is at: *, *P* = 0.03.

To further corroborate the bioassay and proteomic data, we measured the strain enzyme activities by hydrolyzing assays with different substrates. The casein digestion assay indicated that there was no obvious difference between WT and Δ*MrGAG* in forming the hydrolytic-zone sizes seven days post inoculation (Fig 7A). Regarding the chitinase activity, no statistical difference was observed between strains in terms of the digestion of the colloidal chitin (Fig 7B). However, the lipase activity of Δ*MrGAG* was considerably (*P* = 0.03) reduced when compared with that of the WT assayed with the substrate olive oil (Fig 7C), which is consistent with the down-accumulation of the extracellular lipase in proteomic analysis (S3 Table).

## Discussion

After the EPS GAG was first found from *Aspergillus* decades ago [6], we report in this study that the conservative production of GAG plays either similar or unique biological functions in the insect pathogenic fungus *M*. *robertsii*. It was confirmed that the conserved five-gene cluster is responsible for GAG biosynthesis and modification in this insect pathogen. Considering its patchy distribution in a handful of the ascomycete and even basidiomycete fungal species, it is still hard to deduce the origin of this gene cluster in *Metarhizium* species. The event of horizontal gene transfer (HGT) was possible. For example, besides gene family expansions [40], HGT events have been demonstrated with potential contributions to the adaptation of broad host ranges of the generalist *Metarhizium* species [41–43]. It was also suggested that the cordycepin biosynthetic gene cluster in insect pathogenic fungus *Cordyceps militaris*, a close relative of *Metarhizium*, has been originated by HGT from *A*. *nidulans* [44]. Similar to Aspergilli, the divergent *Metarhizium* species so far with the available genome information all contain the GAG biosynthetic gene cluster, suggesting that the common ancestor of *Metarhizium* species might have obtained these genes before fungal speciation. Likewise, the conserved gene cluster is also present in the genomes of different *Neurospora* species such as *N*. *crassa* and *N*. *tatrasperma* (S1 Table). Overall, the origin and patchy distribution pattern of this exopolymer biosynthesis cluster remain to be investigated in the future.

Similar to the finding in *Aspergillus* [8], we found that no GAG is produced on the resting conidia of *M*. *robertsii*. In addition, the hyphal bodies harvested from insect hemolymph are also not covered by the GAG matrix, indicating that GAG will not be produced by *M*. *robertsii* during invasive growth. The observations were consistent with the results of injection bioassays that there were no obvious differences between WT and Δ*MrGAG* against two examined species of insect hosts. In contrast, different studies have confirmed that GAG is required for the development of invasive aspergillosis by mediating adherence, biofilm formation, masking cell wall β-glucans from the dectin-1 recognition and inducing the production of Interleukin-1 receptor antagonist [10,11,13]. It has also been found that GAG polymer could be covered on platelets to trigger complement activation [45]. For insect pathogens like *M*. *robertsii*, however, fungal cells will switch from filamentous growth to yeast-type budding once enter insect body cavities without cell adherence to host matrixes [29]. The cell coat proteins such as the collagen-like protein Mcl1 or LysM-domain protein will be produced to evade and invade insect host immune defences [36,37]. This kind of anti-immune response may preclude the necessity of GAG production on infecting cells. Nevertheless, the regulation control of GAG production remains to be determined in *M*. *robertsii*. It has been recently found that the cell-wall stress factors such as the Calcofluor white and Conge red at low dosages could promote *A*. *fumigatus* to produce GAG, and the clustered genes is regulated by the yeast Flo8/Som1-like transcription factor SomA [46,47]. The SomA-like regulator is also present in *M*. *robertsii* (MAA_00351; 30% identity at amino acid level to the SomA of *A*. *fumigatus*). Future effort(s) is still required to determine whether this putative transcription factor and or other factors are involved in the control of GAG biosynthesis in *Metarhizium* species.

As aforementioned, the exact biosynthetic mechanism of the heteropolysaccharide GAG has not been fully elucidated. We found that the SBA staining signals and mycelium surface matrixes could still be detected on the mutant cells of Δ*MrAgd* and Δ*MrEga*. In addition, similar to the WT, mycelium pellets could also be formed by these two null mutants (Fig 2). Agd3 and Ega3 are functionally linked to each other; the former contributes to GAG deacetylation while the later mediates the disruption of the deacetylated GAG [11,12,25]. Consistent with the findings in *A*. *fumigatus* [11,48], we found that the GAG produced by *M*. *robertsii* is partially deacetylated. Similar to Ega3 [25], MrEga also contains a GH114 domain that may involve in degradation of deacetylaed GAG. Thus, both MrAgd and MrEga may contribute to the structural modification or cleavage of GAG instead of biosynthesis. Even both mutants could still form mycelium pellets like WT in liquid culture, we found that the phenotype of the cell surface GAG matrixes was different between WT and mutants, especially for the appressorium mucilaginous matrixes. In addition, the findings that deletion of either *MrAgd* or *MrEga* could significantly reduce fungal virulence would suggest that aberrant structure of GAG might not be fully functional, which could be supported to some extend that both Δ*MrAgd* and Δ*MrEga* lost their abilities to adhere the hydrophobic surface and form biofilms. Thus, it is functionally required for the deacetylation of GAG to a proper degree. In contrast to Δ*MrAgd*, the *A*. *fumigatus* Δ*Agd3* cells could not be positively stained by SBA and lost the GAG-dependent surface decorations, which indicates that partial deacetylation of GAG is required for the polymer to adhere to fungal cell walls [11,12]. Our genome survey indicated that, in contrast to the absence of *Agd3* homolog in *A*. *fumigatus*, a highly similar paralog of *MrAgd* is present in *M*. *robertsii* (MAA_08880; 64% identity at amino acid level and both contain the Agd3-like CBM87 domain) [49]. The complementary effect of this gene on *MrAgd* deletion is likely to compensate the partial deacetylation of GAG in Δ*MrAgd*, which led to the positive SBA staining and GAG-associated cell wall decorations. Surely, the function of this gene requires further investigations in the future.

An atomic force microcopy study revealed the multifunctional adhesion properties of GAG including the promotion of fungal adhesion to hydrophobic substrates [50]. In this respect, it is not surprising to find that deletion of the gene cluster or any single gene substantially reduced fungal adherence ability to insects, one of the major factors to initiate and determine fungal topical infection. In addition, the mucilaginous matrix formed around the appressoria has long been considered as the typical feature of appressorium formation in support of not only cell attachment but also the secretion and function of cuticle-degrading enzymes such as the serine proteases of *Metarhizium* species [33,34,51]. In this study, we found that GAG production was *de facto* responsible for the appressorial mucilage production in *Metarhizium*, which therefore resolved a long-standing biological puzzle of mucilage formation. Similar to *Metarhizium* species, the rice blast fungus *Magnaporthe oryzae* also produces appressoria along with mucilage adhesives for penetration of host cuticles [52]. It has been recently found that the mucilage contents produced by *M*. *oryzae* could help seal the appressorial pore to facilitate the buildup of appressorial turgor pressure [53]. In this respect, the penetration impairment of Δ*MrGAG* and individual gene-deletion mutants might be additionally due to the defect in generating proper cellular turgor pressure, which likely resulted from the failure of mucilage production for pore sealing instead of lipid-droplet degradation. Intriguingly, however, both our and previous genome surveys indicated that the GAG biosynthetic gene cluster is not present in *M*. *oryzae* [11]. Nevertheless, the conserved cluster is present in the appressorium-producing plant pathogenic fungi such as *Botrytis cinerea* and *Sclerotinia sclerotiorum* (S1 Table) that can also produce appressorium mucilage matrixes [54]. Future efforts are still required to determine the association of GAG production with appressorium mucilage formation in different fungi.

Formation of mycelium pellets is one of the hurdles that can negatively affect the fermentation efficacy of industrial fungi for biomaterial productions [20,55]. It was previously considered that fungal mycelium pellets was formed by the winding of hyphae to form aggregated and compact pellets [56]. We found that GAG could glue the germ tubes together to form hyphal pellets when growing *M*. *robertsii* in liquid culture, which is similar to the finding of *A*. *oryzae* [19]. It was also found in *A*. *nidulans* that deletion of the genes involved in the biosynthesis of the cell wall component α-1,3-glucan could block the formation of mycelium pellets [57]. We found that loss of the exopolymer GAG production did not obviously affect the constitution of other cell wall components of *M*. *robertsii*. It remains to be investigated for *Metarhizium* species whether other components like α-1,3-glucan could affect the formation of mycelium granules.

We found that loss of GAG production could result in differential expression of an array of proteins during *Metarhizium* appressorial formation. However, except for the reduction of total lipase activity, Δ*MrGAG* had similar hydrolytic abilities like the WT against the casein and chitin substrates, which could be due to the cases of both up- and down-accumulation of peptidases and chitinases. Additional biochemical effects of GAG remain to be investigated in the future.

In conclusion, we report the biosynthesis and function of GAG in the insect pathogenic fungus *M*. *robertsii*. In particular, we found that GAG is responsible for the formation of appressorium mucilaginous matrix and penetration of insect cuticles, the unique biological effects of GAG that are absent from *Aspergillus* species. Considering that the conserved biosynthetic gene cluster is also present in the genomes of fungi with different lifestyles such as the saprophyte *N*. *crassa* and the nematode-trapping fungus *A*. *oligospora*, biosynthesis and unique biological potentials of GAG require further investigations in these non-*Aspergillus* fungi.

## Materials and methods

### Fungal strains and maintenance

The WT strain *M*. *robertsii* strain ARSEF 23 and its derived mutants were cultured on PDA (BD Difco) at 25°C. For genomic DNA and RNA extractions, spore germination and hyphae staining, fungal spores were inoculated in SDB (Bd Difco) and incubated for different times at 25°C and 200 rpm in a rotary shaker. Appressorium formations were induced either on cicada (*Cryptotympana atrata*) wings, front wings of the mealworm *Tenebrio molitor* or on petri dish hydrophobic surfaces containing the minimal medium (MM; NaNO_3_, 6 g/l; KCl, 0.52 g/l; MgSO_4_·7H_2_O, 0.52 g/l; KH_2_PO_4_, 0.25 g/l) amended with 1% glycerol as the sole carbon resource [32]. Appressoria of the WT and mutant induced on hydrophobic surfaces were stained with the fluorescent dye Bodipy (Thermo Fisher Scientific) to determine the distribution and degradation of lipid droplets [58]. For stress challenges, the WT and mutant strains were grown on MM agar, PDA or PDA amended with the final concentrations of 0.15 M LiCl, 0.5 M sorbitol and 50 μg/ml Congo red for osmotic and cell wall integrity challenges [59].

### Gene deletion and complementation

Deletion of the whole cluster or individual genes was performed in the WT strain of *M*. *robertsii* via *Agrobacterium*-mediated transformation [60]. In brief, the 5’- and 3’-flanking sequences of each and the whole cluster were amplified using the genomic DNA as a template with different primer pairs (S5 Table). The PCR products were digested with the restriction enzymes and then inserted into the same-enzyme treated binary vector pDHt-bar (conferring resistance against ammonium glufosinate) to produce the corresponding plasmid for fungal transformation. The drug resistance colonies were verified by PCR using the genomic DNA as templates and different primers (S5 Table). The putative null mutants were used for single spore isolations and at least five clones of each mutant were then grown in SDB for 36 hrs. The mycelia of each strain were harvested for RNA extraction and RT-PCR verification of gene deletions. At least three independent mutants were selected for each gene deletion and one without phenotype differences from each other was randomly selected for further experiments. The confirmed null mutants Δ*MrAgd*, Δ*MrSph* and Δ*MrUge* were selected for gene complementation. Thus, the corresponding gene was amplified together with its promoter region (ca. 1.5 kb upstream of the start codon) using different primers (S5 Table). The purified fragments were individually cloned into the vector pDHt-sur (with a *sur* gene for conferring sulfonylurea resistance) [61]. The null mutants were then transformed to obtain the drug-resistant colonies, which were further verified by PCR and RT-PCR for successful gene complementation.

### GAG extraction and quantification

To determine the effect of gene deletions on GAG biosynthesis, we extracted GAG by ethanol precipitation [8,11]. Both the WT and Δ*MrGAG* spores were inoculated in SDB with three replicates each for three days. The mycelia of each replicate were dried overnight at 65°C and weighted. The culture filtrates were collected by filtration and precipitated with 2.5 volume of ethanol for overnight at 4°C. The filtrate samples were then centrifuged at 3,000 g for 10 min and the pellets were washed twice with 150 mM NaCl. After centrifugation, the pellets were then extracted with 8 M urea twice (2 h for each treatment) under shaking at room temperature. The supernatant of each sample were pooled, dialyzed against water and freeze dried as urea-soluble (US) parts. Urea-insoluble (UI) pellets were washed with water twice and freeze-dried. In addition, both US and UI samples were subjected to acid hydrolysis with 4 N HCl for 4 hr at 100°C. The samples were then analyzed by gas chromatography-mass spectrometry (GC-MS; Agilent, Santa Clara, CA) for relative quantification of galactose within each sample [8]. There were three replicates for each strain and the differences were compared between WT and Δ*MrGAG* by the two-tailed Student’s *t*-test.

### Analysis of GAG acetylation

To determine the (de)acetylation degree of GAG produced by *M*. *robertsii*, we grew the WT and Δ*MrAgd* in SDB for three days. The culture filtrates were precipitated with ethanol, washed with NaCl, dialyzed and freeze dried. The samples (3 mg each) were then hydrolyzed in 4 M HCL (0.6 ml) at 100°C for 4 h and each sample was divide into two aliquots. One part was dried overnight in a desiccator and dissolved in sterile water for MBTH (3-Methyl-2-benzothiazolinon hydrazine hydrochloride hydrate, Anpel) assays for quantification of aldehydes (reducing sugars) [48,62]. The reaction was carried in a 96-well plate and measured with the Multiskan FC microplate reader (Thermo Scientific, Waltham, MA). A calibration curve was generated using a serial dilution of GalNAc (Aladdin). The other part acidic hydrolytic samples (300 μl each) were neutralized with 7 M NaOH and 2 M MOPS [3-(N-morpholino) propanesulfonic acid] and then subjected to the enzymatic acetate (a product of deacetylation) assay with the Acetate Colorimetric Assay Kit (Sigma-Aldrich, St. Louis, MO). The GalNAc sample was used as a control. The acetate content with each sample was calibrated with the standard solution by following the manufacturer’s protocol. There were three replicates for each sample. The acetylation level of each sample was expressed as acetate per unit reducing sugar. To further confirm the constituent of the GAG produced by *M*. *robertsii*, we performed liquid chromatography (LC)-MS analysis (UltiMate 3000 BioRS System, Thermo Scientific) of the hydrolytic WT and Δ*MrAgd* samples together with the standards galactose (SinoPharm), GalN (Aladdin), GalNAc and acid-hydrolyzed GalNAc.

### Fluorescent staining

For detecting the production of GAG, the GalNAc specific lectin SBA was used for staining of the WT and mutant cells using the fluorescein conjugated SBA (Vector Lab) [10]. Fungal conidia were harvested from the two-week old PDA plates, and germinated in SDB for 16 hrs in a rotary shaker at 25°C prior to SBA staining. As indicated above, appressoria were induced on petri dishes. Fungal hyphal bodies were prepared by injecting the last instar larvae of the wax moth *Galleria mellonella* with spore suspensions (10 μl each of 1 × 10^6^ conidia/ml) at the second proleg. Insect hemolymph was collected on ice 60 hrs post injection and used for gradient centrifugation in percoll at 4° C and 5500 rpm for 10 min [36].

To determine the effect of GAG loss on the distribution/accumulation of other cell wall components, we used different fluorescent lectins to stain and detect the polysaccharides on fungal germlings. These lectins included ConA (for detecting α-mannopyranosyl and α-glucopyranosyl residues), GSII (*Griffonia simplicifolia* lectin II for detecting α- or β-linked *N*-acetyl-*D*-glucosamine), and GNL (*Galanthus nivalis* lectin for detecting α-1,3-mannose) (Sigma-Aldrich), which were prepared in different buffers [63]. In addition, the wheat germ agglutinin (WGA-AF488, Thermo Fisher Scientific) was used for selective binding *N*-acetyl-*D*-glucosamine and *N*-acetylneuraminic acid residues [64]. All fungal cells were washed three times with the phosphate buffer solution (PBS, pH 7.0) prior to staining in dark for 1 hr. After washing with PBS for three times, samples were observed under a fluorescent microscopy (Nikon, CX21).

### Scanning and transmission electron microscopy analysis

To determine hyphal surface differences between WT and mutants, we harvested the mycelial samples from the day 3 SDB cultures for the observations with both the Field Emission Scanning Electron Microscopy (Carl Zeiss, Oberkochen, Germany) [65] and Transmission Electron Microscopy (TEM, H-7650; Hitachi) [60]. The harvested mycelia were fixed overnight in 2.5% glutaraldehyde buffered in PBS (pH, 7.2). For SEM analysis, the samples were dehydrated with ethanol gradients (50–100%) and coated with platinum. For TEM observations, the samples were embedded in Epson resin for sectioning and the obtained ultrathin samples were then treated in 2% uranium acetate and lead citrate prior to TEM analysis [58]. In addition, fungal appressoria were induced on mealworm front wings and then subjected to SEM analysis. The conidia of the WT and Δ*MrGAG* harvested from two-week old PDA were also used for SEM analysis.

### Insect bioassays

To determine the effect of GAG biosynthesis on fungal virulence, we performed both topical infection and injection assays using two insect species, i.e., the wax moth *G*. *mellonella* and the fruit fly *D*. *melanogaster*. Fungal spores of the WT and different mutants were harvested from two-week old PDA plates and suspended in 0.05% Tween-20. Spore concentrations were adjusted to 2.5 × 10^7^ conidia/ml for topical immersion (30 sec) assays against the last instar larvae of wax moth. For injection assays of WT and Δ*MrGAG*, each larvae were injected with 20 μl spore suspension (1 × 10^6^ conidia/ml) at the second proleg. There were three replicates with 15 insects per replicate for each strain. The WT and Δ*MrGAG* were also assayed against the female adults of the fruit fly W1118 line (2–5 days post eclosion) for both topical infection (immerged in 1 × 10^6^ conidia/ml suspension for 30 sec) and injection of 5 nl suspension of 1 × 10^7^ conidia/ml per insect with a microinjector (Nanoject III, Drummond, Broomall, PA). There were three replicates with 20 flies per replicate for each strain. The experiments were repeated at least twice. For topical infections, the treated insects were kept at a high moisture (> 98% relative humidity) for the first 24 hrs and then maintained at the room condition. Insect survivals were checked every 12 hrs and the insect survival kinetics were compared by Kaplan-Meier analysis with a Log-rank test [59].

### Spore hydrophobicity, adhesion and penetration assays

To determine conidial spore hydrophobicity, we harvested the WT and Δ*MrGAG* conidia from the two-week old PDA plates. The spores were suspended in sterile water by rigorous vortexing. Spore concentration of the WT and Δ*MrGAG* was adjusted to 1 × 10^8^ conidia/ml and the hydrophobicity index of each sample was then determined based on spore affinity to hexadecane as described previously [65]. Spore adhesion of the WT and Δ*MrGAG* was assayed against both the plastic petri dish and insect body surface. Thus, two different concentrations of spore suspension (1 × 10^5^ conidia/ml and 1 × 10^6^ conidia/ml) were inoculated (20 μl each) into the wells of the Corning 24-well plate (each well pre-added with 200 μl SDB) and incubated at 25°C for 24 hrs. The supernatant was then carefully removed from each well and the wells were washed with PBS for three times prior to the addition of 1 ml of 0.05% crystal violet for 30 min. After washing with PBS for three times, the plate was photographed to indicate the adherence of fungal cells to hydrophobic surface. The variation of the spore binding to insect cuticle was determined between WT and mutant by dichloromethane wash [66]. The female fruit fly adults and last instar larvae of wax moth were immersed in the spore suspension (1 × 10^7^ conidia/ml) for 30 sec, and the insects were then kept at a high moisture condition for 8 hrs prior to wash with 0.05% Tween-20 to remove those unbounded spores. The insects were finally washed with dichloromethane to count the wash-off spores (germlings).

Penetration capacity of the WT and different mutants was assayed using both the cellophane membrane and cicada wings [67]. The sterilized membrane and wings were lined on the surface of MM plates and 2 μl spore suspension (1 × 10^7^ conidia/ml) were inoculated in the middle. After incubation for three days on cellophane membrane and 40 hrs on insect wings, fungal cultures were carefully removed together with the membrane and wings. The plates were continuously incubated for another week to unveil the difference of fungal penetration ability.

### Comparative proteomic analysis

Comparative proteomic analysis was conducted to determine the difference of protein expressions between WT and Δ*MrGAG* during appressorium formation. Thus, appressoria were induced on cicada wings by immersion the wings in the spore suspensions (1 × 10^8^ conidia/ml) for 30 sec prior to the transfer on water agar plates for 24 hrs. The wing samples (1 g) were then finely ground in liquid nitrogen, and the powders were solubilized in 1/10 volume of the buffer (4% SDS, 100 mM DTT, and 150 mM Tris-HCl, pH 8.0). After boiled in water for 3 min, the suspensions were ultrasonicated (80 w, 10 sec per ultrasonic treatment for 10 times) and then boiled again for 3 min. The crude extracts were centrifuged at 13,000 g at 4°C for 10 min. The supernatants were collected and protein content of each sample was determined using the Protein Assay Reagent (Promega, USA). The protein samples were individually digested with trypsin and each sample (100 μg) was then labelled with an isobaric tag (tandem mass 115–119), pooled and subjected to iTRAQ (isobaric Tags for Relative and Absolute Quantitation) proteomic analysis with a Q-Exactive Hybrid Quadrupole-Orbitrap LC-MS/MS system (Thermo Fishser). Ion score of each fragment was processed with MASCOST (ver. 2.2; Matrix Science) and the data were then searched using the Proteome Discoverer program (ver. 1.4; ThermoFisher) against the whole genome protein data of *M*. *robertsii* [40]. Ratiometric normalization was performed using the signal intensity data to center the ratio distribution on 1 [68]. There were three biological repeats for each strain. Full MS data have been deposited at the PRIDE database [69] with the identifier accession PXD019301. A protein (with at least one unique peptide being detected twice) was considered differently expressed between strains based on the Student’s *t-*test *P* ≤ 0.01 and FDR (false discovery rate) ≤ 0.05 [70]. The detected GAG biosynthetic protein, if any, was only considered being expressed by the WT.

To verify protein differential expression, we performed a PRM analysis [39]. In brief, each digested peptide sample was mixed with an equivalent iRT-Kit peptide (Biognosys) as an internal standard. For each analysis, 2 μl of each sample was loaded and the mass spectrometer was operated in a positive ion mode. Full MS scans were followed by 49 PRM scans at 35,000 resolution (at 200 m/z) as triggered by a schedule inclusion list. All PRM data analysis and integration were performed with the Skyline software (3.5.0) [71]. Three independent biological replicates were performed for each sample. The significance of difference was determined by a two-tailed Student’s *t*-test at a level of *P* < 0.05.

### Enzyme hydrolytic assays

To determine the difference of the protease, chitinase and lipase activities between WT and Δ*MrGAG*, we conducted hydrolytic zone assays. Thus, fungal cultures (mycelium mats each of 5 mm diameter) were co-inoculated on the MM agar containing 1% (w/v) casein for 7 days [59]. For chitinase activity assays, both WT and Δ*MrGAG* were inoculated on the MM agar containing 1% (w/v) colloidal chitin (Sigma-Aldrich) for 7 days [72]. Plate lipase activities were assayed using the substrate olive oil (1%, v/v) plus the indicator agent bromcresol purple (0.2%, w/v) [73]. Colony diameters were measured and the difference of hydrolytic zone formation was analyzed between WT and Δ*MrGAG* by a two-tailed Student’s *t*-test.

## Supporting information

S1 Fig Verification of gene deletions. (A) PCR verification. Genomic DNA of the WT and mutants were extracted and used as templates for PCR verification. (B) RT-PCR verification. Mycelia of the WT and mutants were harvested from the day 3 SDB for RNA extraction and RT-PCR analysis.
(TIF)

S2 Fig Phenotyping of the WT and mutant strains on different media. (A) No obvious difference of the growth and sporulation on PDA between WT and mutants. (B) No obvious difference of stress responses between WT and Δ*MrGAG*.
(TIF)

S3 Fig LC-MS analysis of the GAG-associated monosaccharides. (A) Presence of galactose (Gal) in the hydrolytic EPS samples of WT and Δ*MrAgd*. (B) Presence of GalN in the hydrolytic EPS samples of WT and Δ*MrAgd*, and the hydrolyzed GalNAc. (C) Non-presence of GalNAC in the hydrolytic EPS samples of WT and Δ*MrAgd*, and the hydrolyzed GalNAc. EIC, extracted ion chromatogram. The standards Gal, GalN, GalNAc and hydrolyzed GalNAc were included as reference controls.
(TIF)

S4 Fig Gene complementation and mutant phenotyping. (A) Positive SBA staining after gene complementation (CP) of the individual gene deletion mutants. Bar, 5 μm. (B) Mycelium pellet production by the gene-complemented mutants. (C) Variation of the spore adhesion ability between different null and gene-complementation mutants toward hydrophobic surface. Spore suspensions (each at a final concentration of 1 × 10^6^ conidia/ml) were inoculated into the 24-well plate for 24 hrs and then washed off with PBS buffer. The wells were then stained with crystal violet before imaging.
(TIF)

S5 Fig Association of GAG production with mycelial pellet formation. Conidial spores were inoculated in SDB for 6 hrs (A), 9 hrs (B) and 12 hrs (C), germling aggregation and mycelial pellet formation could be evident for the WT but not for Δ*MrGAG*. After SBA staining, GAG could be detected on the WT cells but not on mutants (lower panels of panel C). Bar, 10 μm.
(TIF)

S6 Fig Conidial surface phenotyping and fungal penetration assays. (A) SEM observation of the WT and mutant conidial surface. Bar, 200 nm. For penetration assays, both the WT and individual gene deletion mutants were inoculated on cellophane for 3 days (B) or cicada wings for 40 hrs (C). The cellophane and insect wings were then carefully removed with fungal cultures and the plates were kept for incubation for one week.
(TIF)

S7 Fig Quantification and comparison of the hyphal body formation in insect hemocoels between WT and Δ*MrGAG*. (A) Microscopic observation of the hyphal-body cells (arrowed) in insect hemolymph. Bar, 15 μm. (B) Quantification of hyphal bodies formed in insect hemolymph. The last instar larvae of the wax moth were bled for microscopic observation 72 hrs post topical infection by WT and mutant. The significance of the two-tailed Student’s *t*-test difference is at: **, *P* = 0.0063.
(TIF)

S8 Fig Comparative proteomic analysis of the WT and Δ*MrGAG* appressorial samples. (A) Protein gel profiling of the three independent samples extracted from the WT and Δ*MrGAG* appressoria formed on the cicada wings. (B) Volcano plotting of the proteomic data. Blue and red spots showing the differentially expressed proteins. (C) FunCat analysis of the proteins upregulated in Δ*MrGAG* when compared with those of the WT strain. (D) FunCat analysis of the proteins down-accumulated in Δ*MrGAG* when compared with those of the WT strain.
(TIF)

S9 Fig Heat mapping analysis of protein differential expressions between WT and Δ*MrGAG*. (A) Heat mapping of the WT and Δ*MrGAG* appressorial protein expression profiles. The proteins were selected for heat mapping analysis based on their expressional difference between WT and mutant with cut-off values of the Student’s *t-*test *P* ≤ 0.01 and FDR ≤ 0.05. (B) Differential expression of the selected proteins/enzymes detected in the WT and Δ*MrGAG* appressoria. GHs, glycoside hydrolases; CW, cell wall. Fungal appressoria were induced on cicada wings for 24 hrs and proteins were then extracted for proteomic analysis.
(TIF)

S1 Table Presence of the conserved GAG biosynthetic gene cluster in different fungi. (PDF)

S2 Table Estimation and comparison the median lethal time (LT_50_, hours) between WT and different mutants during topical infection of the wax moth larvae. (PDF)

S3 Table Differential protein expressions between WT and Δ*MrGAG* during appressorial formation. (XLSX)

S4 Table Verification and comparison of the selected protein expressions detected by PRM and iTRAQ analyses. (PDF)

S5 Table PCR primers used in this study. (PDF)
