# Supplementary figures and images for: Conservative production of galactosaminogalactan in Metarhizium is responsible for appressorium mucilage production and topical infection of insect hosts
Source: PLoS Pathog. 2021 Jun 14;17(6):e1009656. doi: 10.1371/journal.ppat.1009656 (PMC8224951; doi:10.1371/journal.ppat.1009656)

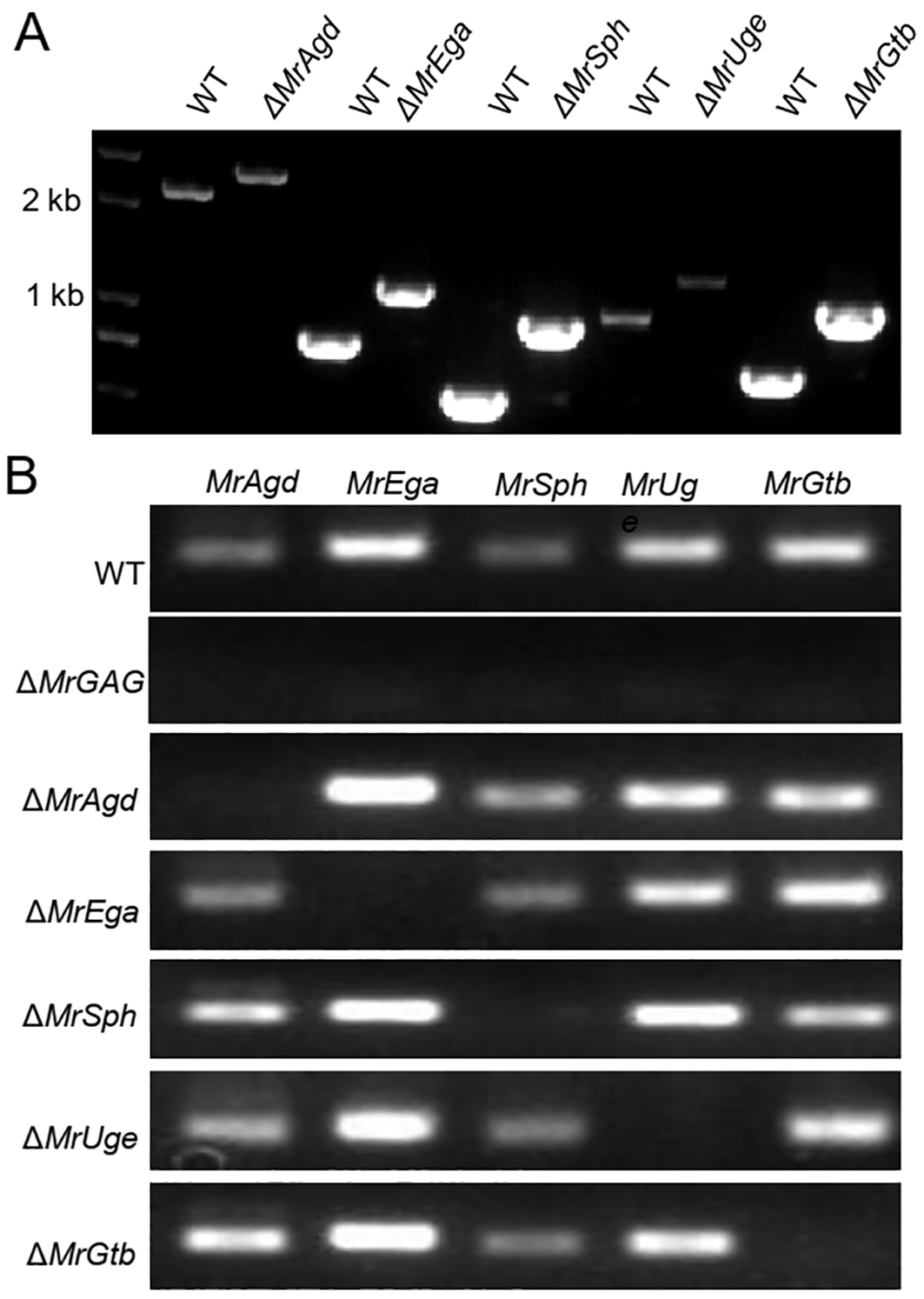

Supplement: S1 Fig — (A) PCR verification. Genomic DNA of the WT and mutants were extracted and used as templates for PCR verification. (B) RT-PCR verification. Mycelia of the WT and mutants were harvested from the day 3 SDB for RNA extraction and RT-PCR analysis. (TIF) [file ppat.1009656.s001.tif]

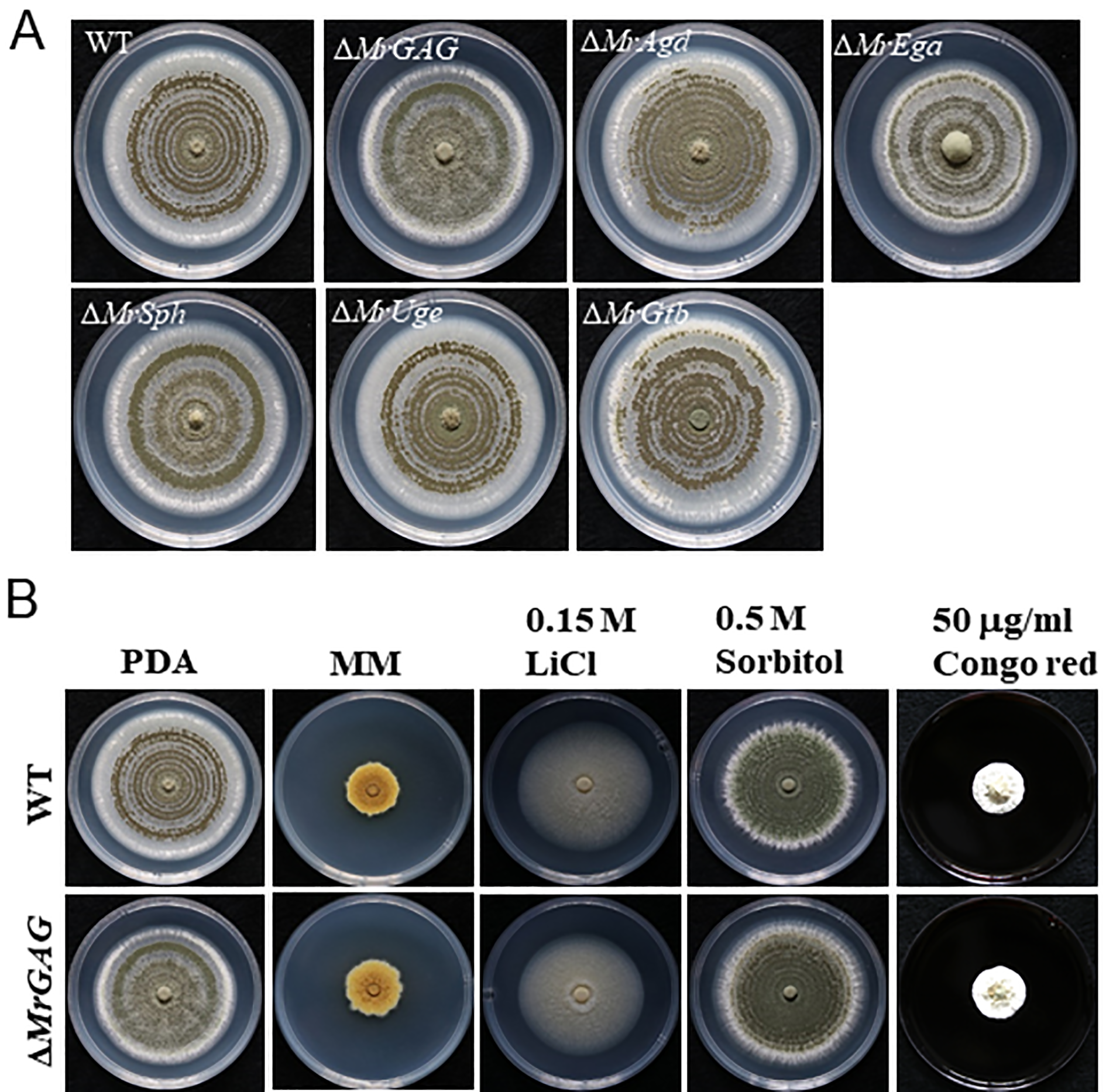

Supplement: S2 Fig — (A) No obvious difference of the growth and sporulation on PDA between WT and mutants. (B) No obvious difference of stress responses between WT and ΔMrGAG. (TIF) [file ppat.1009656.s002.tif]

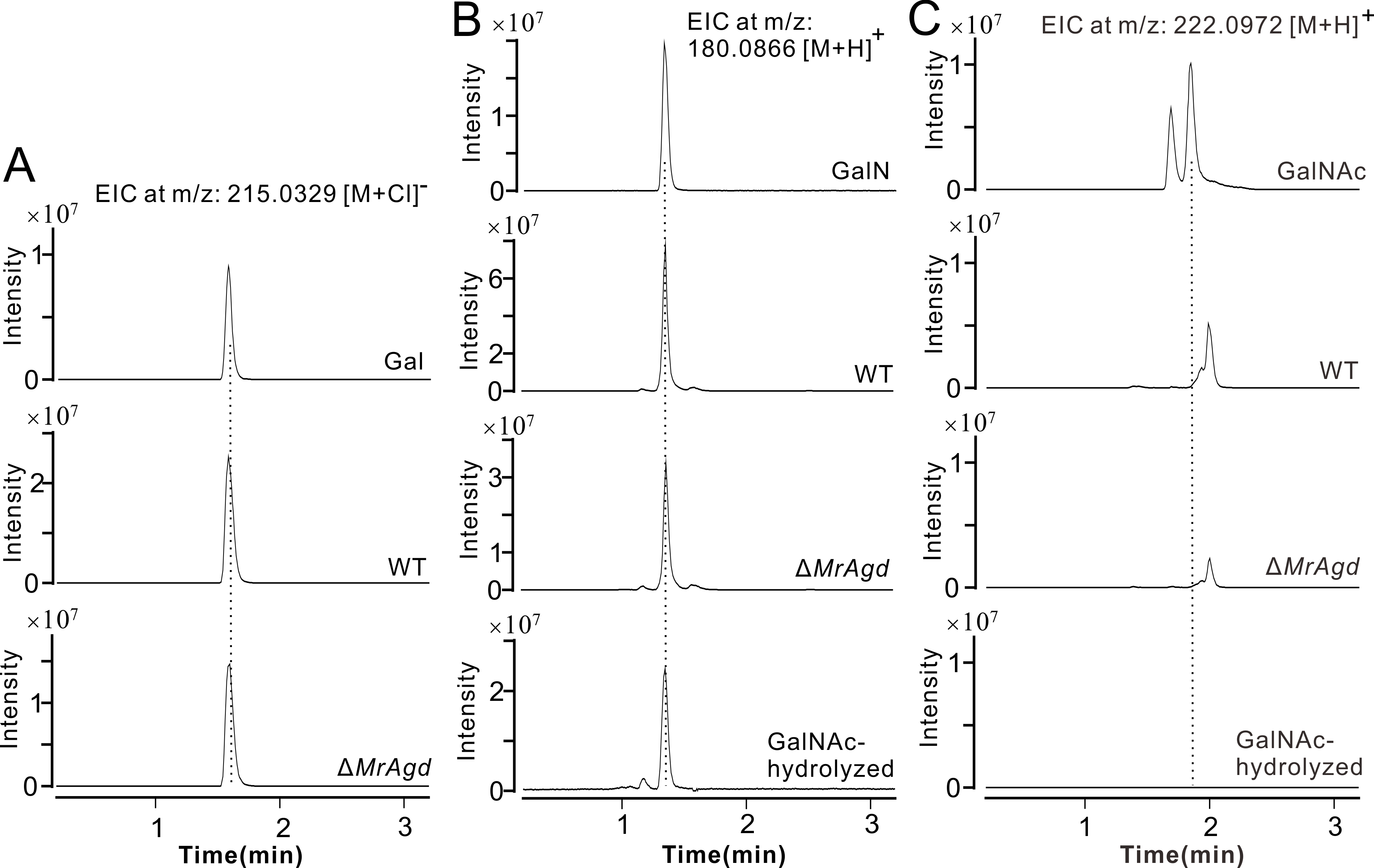

Supplement: S3 Fig — (A) Presence of galactose (Gal) in the hydrolytic EPS samples of WT and ΔMrAgd. (B) Presence of GalN in the hydrolytic EPS samples of WT and ΔMrAgd, and the hydrolyzed GalNAc. (C) Non-presence of GalNAC in the hydrolytic EPS samples of WT and ΔMrAgd, and the hydrolyzed GalNAc. EIC, extracted ion chromatogram. The standards Gal, GalN, GalNAc and hydrolyzed GalNAc were included as reference controls. (TIF) [file ppat.1009656.s003.tif]

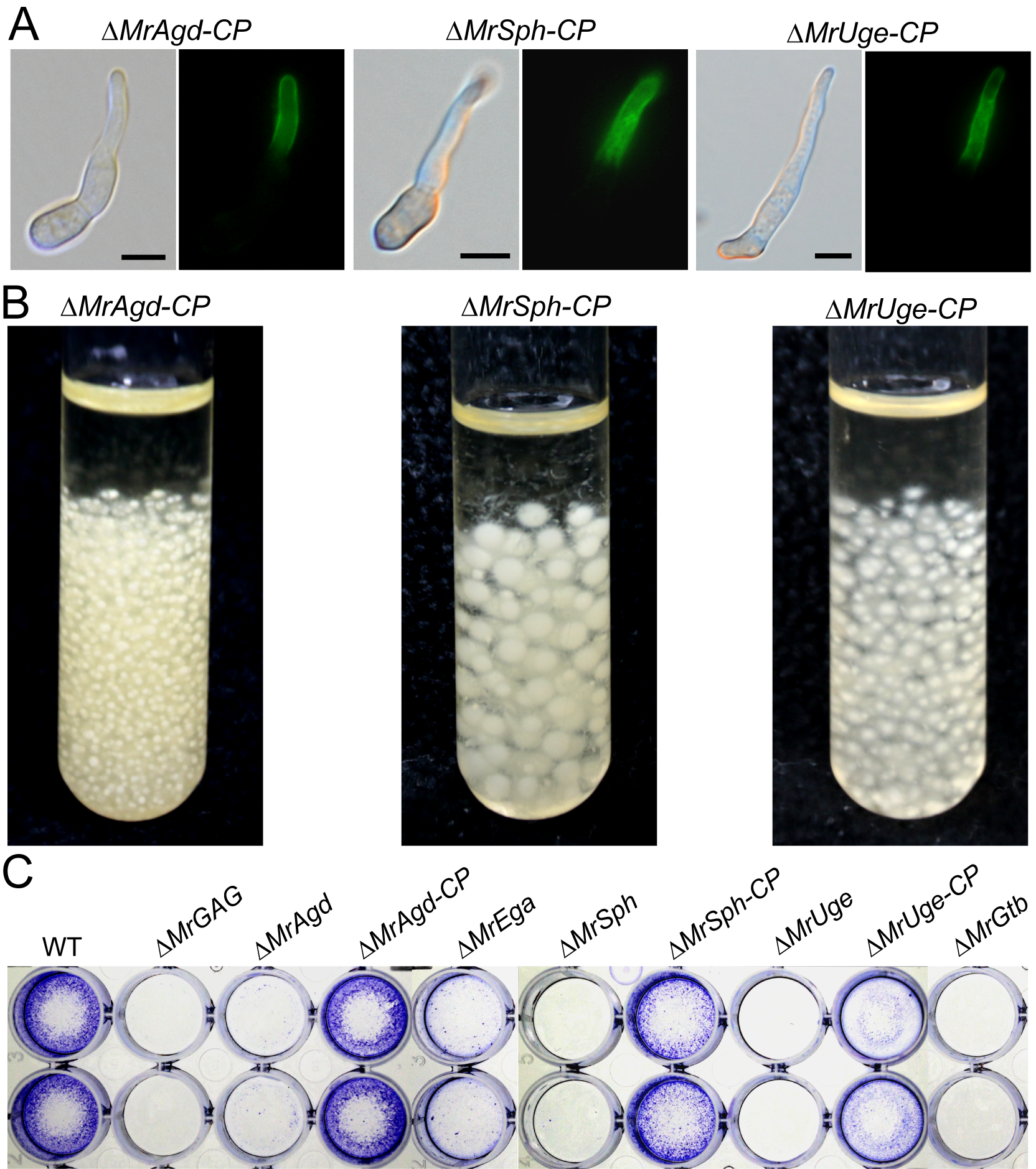

Supplement: S4 Fig — (A) Positive SBA staining after gene complementation (CP) of the individual gene deletion mutants. Bar, 5 μm. (B) Mycelium pellet production by the gene-complemented mutants. (C) Variation of the spore adhesion ability between different null and gene-complementation mutants toward hydrophobic surface. Spore suspensions (each at a final concentration of 1 × 10^6^ conidia/ml) were inoculated into the 24-well plate for 24 hrs and then washed off with PBS buffer. The wells were then stained with crystal violet before imaging. (TIF) [file ppat.1009656.s004.tif]

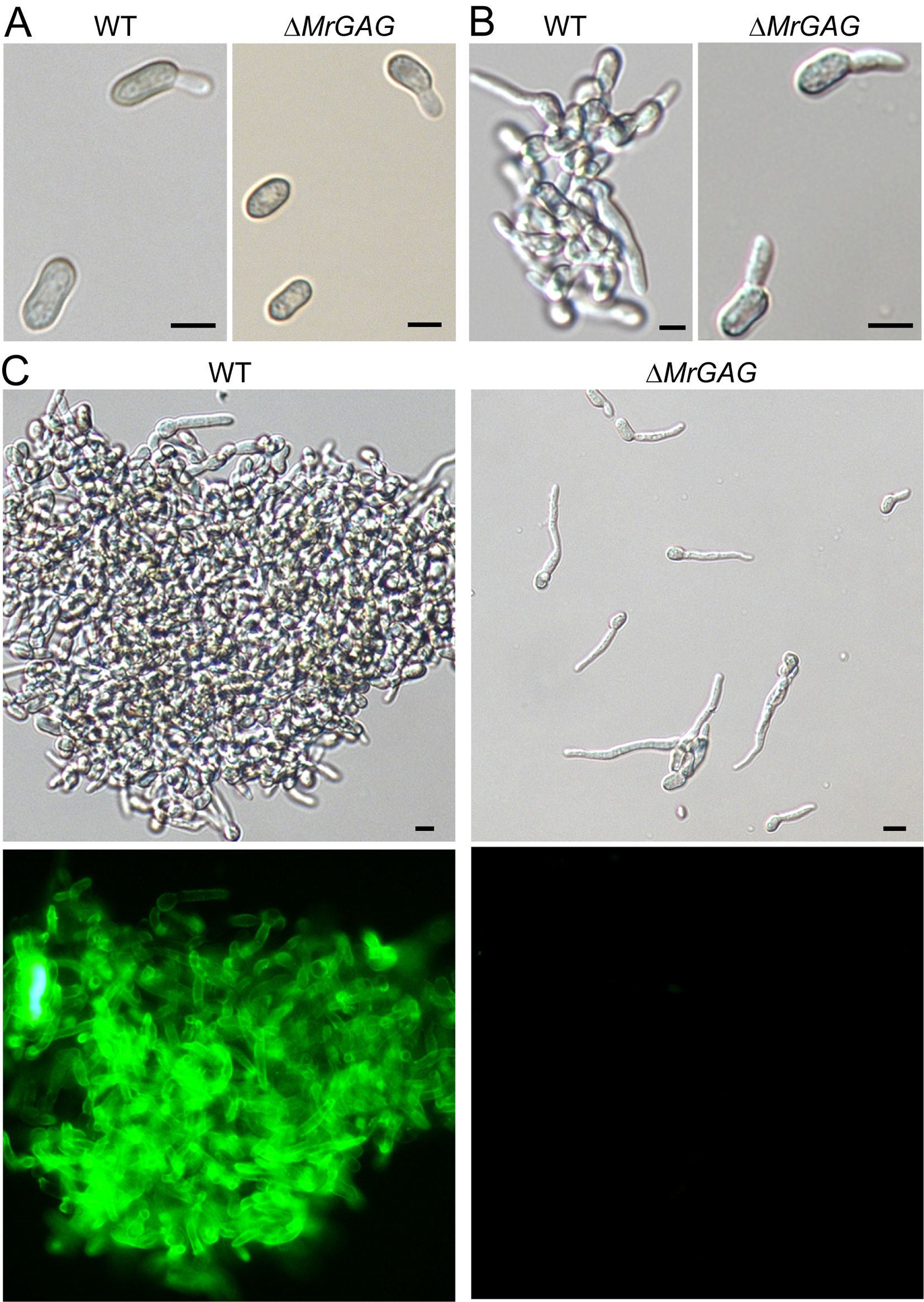

Supplement: S5 Fig — Conidial spores were inoculated in SDB for 6 hrs (A), 9 hrs (B) and 12 hrs (C), germling aggregation and mycelial pellet formation could be evident for the WT but not for ΔMrGAG. After SBA staining, GAG could be detected on the WT cells but not on mutants (lower panels of panel C). Bar, 10 μm. (TIF) [file ppat.1009656.s005.tif]

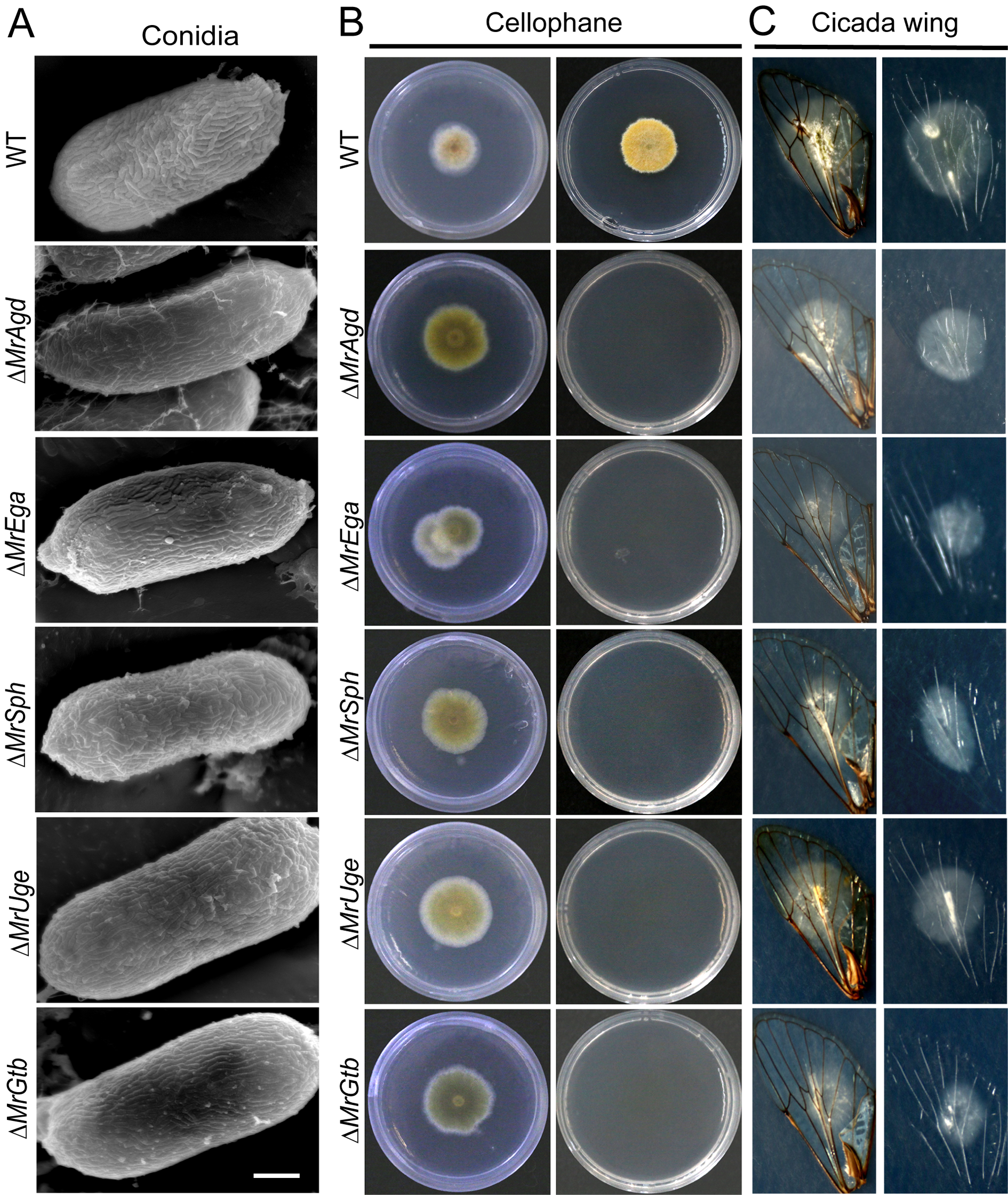

Supplement: S6 Fig — (A) SEM observation of the WT and mutant conidial surface. Bar, 200 nm. For penetration assays, both the WT and individual gene deletion mutants were inoculated on cellophane for 3 days (B) or cicada wings for 40 hrs (C). The cellophane and insect wings were then carefully removed with fungal cultures and the plates were kept for incubation for one week. (TIF) [file ppat.1009656.s006.tif]

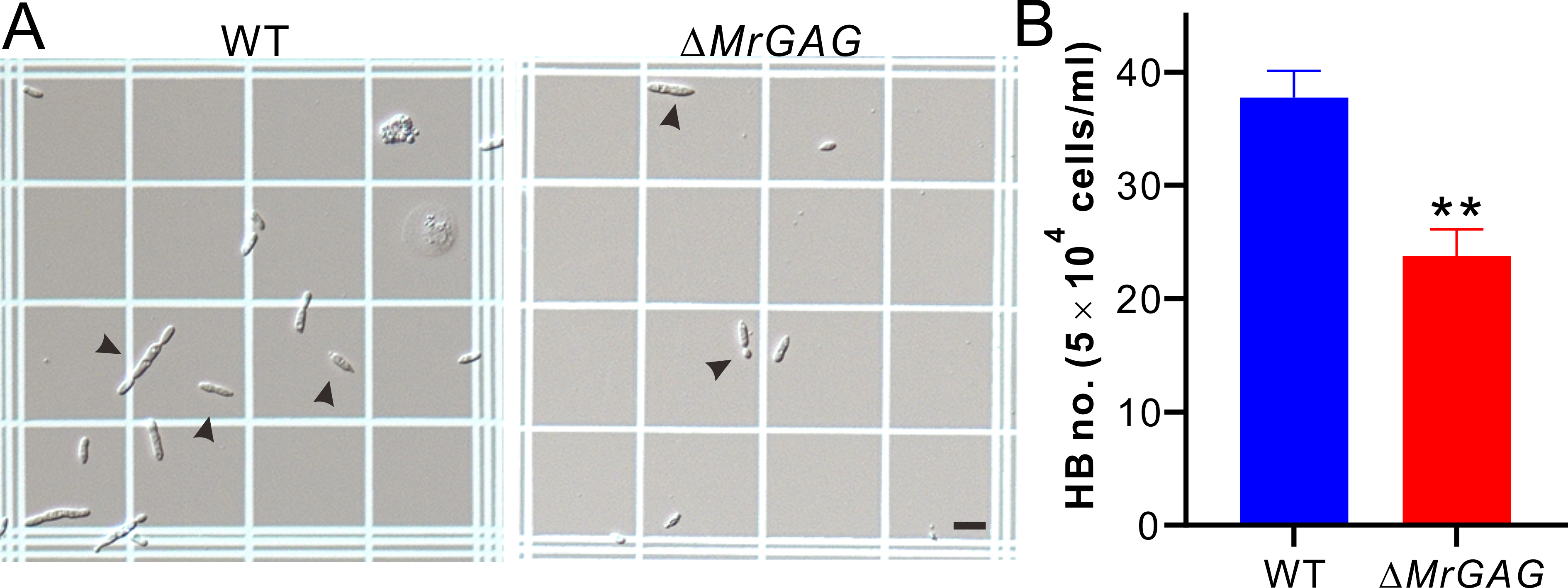

Supplement: S7 Fig — (A) Microscopic observation of the hyphal-body cells (arrowed) in insect hemolymph. Bar, 15 μm. (B) Quantification of hyphal bodies formed in insect hemolymph. The last instar larvae of the wax moth were bled for microscopic observation 72 hrs post topical infection by WT and mutant. The significance of the two-tailed Student’s t-test difference is at: **, P = 0.0063. (TIF) [file ppat.1009656.s007.tif]

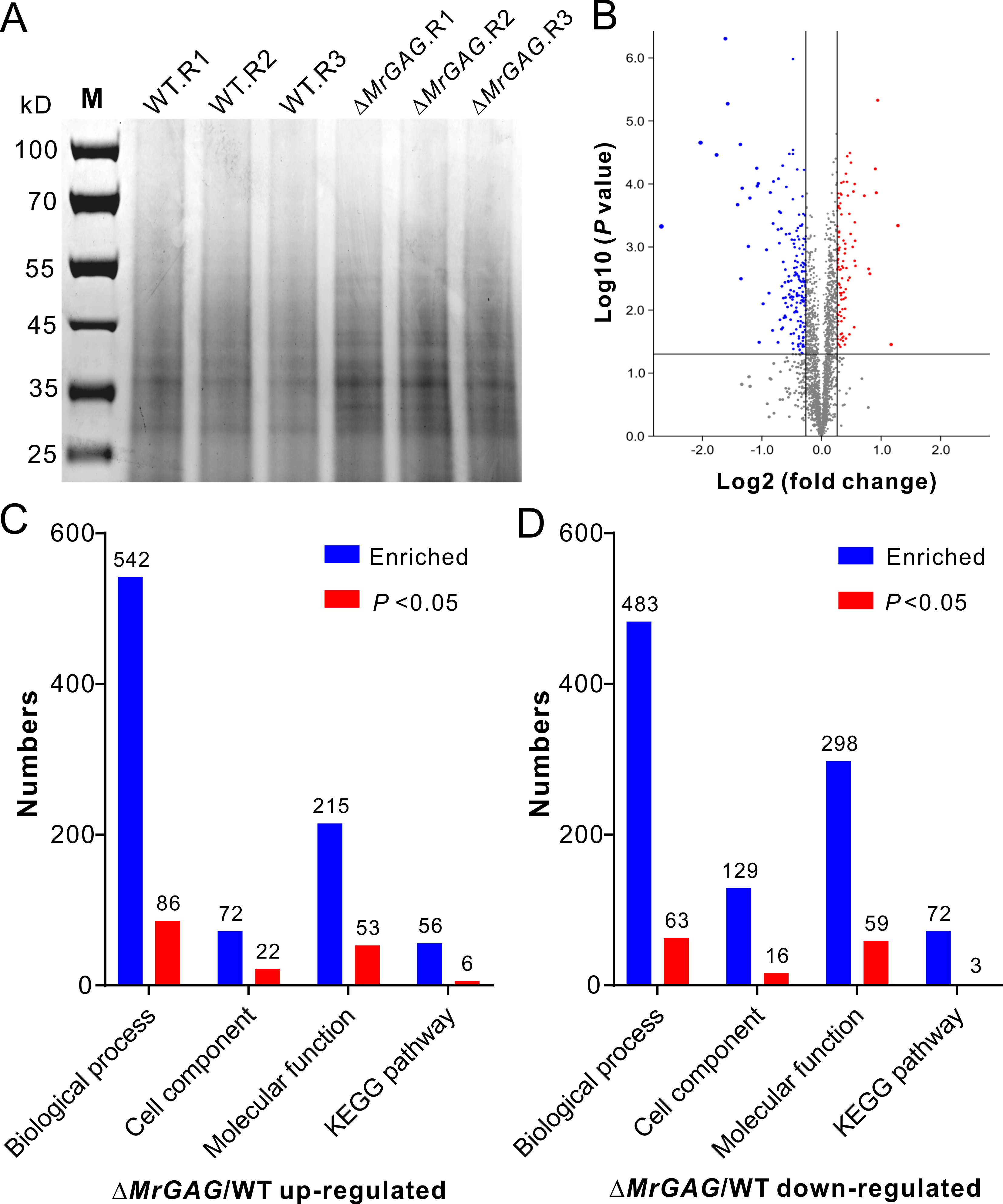

Supplement: S8 Fig — (A) Protein gel profiling of the three independent samples extracted from the WT and ΔMrGAG appressoria formed on the cicada wings. (B) Volcano plotting of the proteomic data. Blue and red spots showing the differentially expressed proteins. (C) FunCat analysis of the proteins upregulated in ΔMrGAG when compared with those of the WT strain. (D) FunCat analysis of the proteins down-accumulated in ΔMrGAG when compared with those of the WT strain. (TIF) [file ppat.1009656.s008.tif]

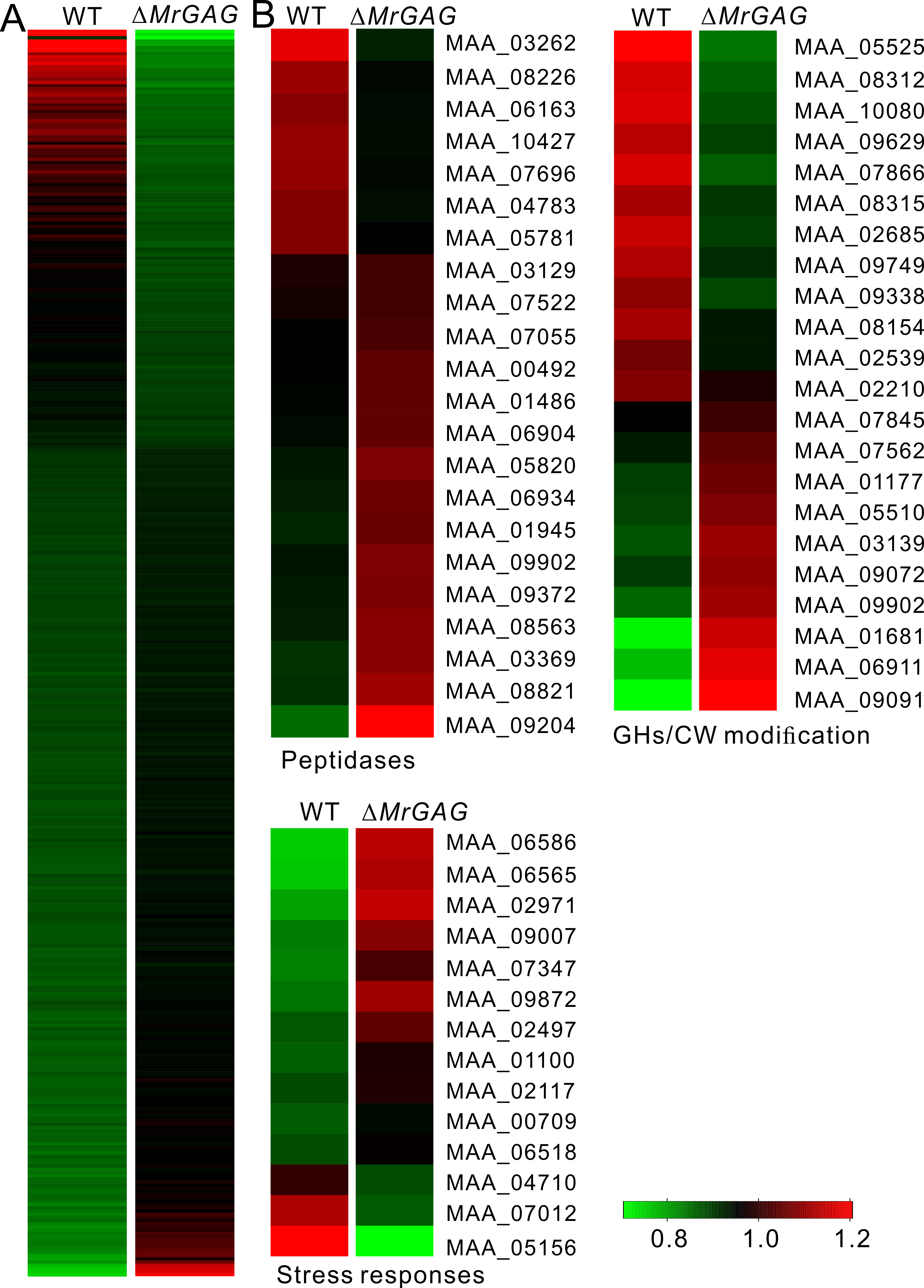

Supplement: S9 Fig — (A) Heat mapping of the WT and ΔMrGAG appressorial protein expression profiles. The proteins were selected for heat mapping analysis based on their expressional difference between WT and mutant with cut-off values of the Student’s t-test P ≤ 0.01 and FDR ≤ 0.05. (B) Differential expression of the selected proteins/enzymes detected in the WT and ΔMrGAG appressoria. GHs, glycoside hydrolases; CW, cell wall. Fungal appressoria were induced on cicada wings for 24 hrs and proteins were then extracted for proteomic analysis. (TIF) [file ppat.1009656.s009.tif]
