# Supplementary material for: Conservative production of galactosaminogalactan in Metarhizium is responsible for appressorium mucilage production and topical infection of insect hosts
Source: PLoS Pathog. 2021 Jun 14;17(6):e1009656. doi: 10.1371/journal.ppat.1009656 (PMC8224951; doi:10.1371/journal.ppat.1009656)
Supplement: S1 Table — (PDF) [file ppat.1009656.s010.pdf]

S1 Table. **Presence of the conserved GAG biosynthetic gene cluster in different fungi.**

**Fungal species**	**GAG biosynthetic gene cluster**				
A. fumigatus	Afu3g07870 (*agd3*)	Afu3g07890 (*ega3*)	Afu3g07900 (*sph3*)	Afu3g07910 (*uge3*)	Afu3g07860 (*gtb3*)
A. nidulans	AN2954	AN2953	AN2952	AN2951	AN2955
M. robertsii	MAA_06941 (*MrAgd*; 48%*)	MAA_06942 (*MrEga*; 47%)	MAA_06943 (*MrSph*; 34%)	MAA_06944 (*MrUge*; 51%)	MAA_06945 (*MrGtb*; 28%)
M. anisopliae	MAN_07185	MAN_07186	MAN_07187	MAN_07188	MAN_07189
M. brennum	MBR_09142	MBR_09141	MBR_09140	MBR_09139	MBR_09138
M. guizhouense	MGU_09803	MGU_09802	MGU_09801	MGU_09800	MGU_09799
M. majus	MAJ_08446	MAJ_08447	MAJ_08448	MAJ_08449	MAJ_08450
M. rileyi	NOR_03856	NOR_03857	NOR_03858	NOR_03859	NOR_03860
M. acridum	MAC_02143	MAC_02142	MAC_02141	MAC_02140	MAC_02139
M. album	MAM_08305	MAM_08304	MAM_08303	MAM_08302	MAM_08301
Neurospora crassa	NCU05137	NCU05136	NCU05135	NCU05133	NCU05132
Neurospora tetrasperma	XP_009854704	XP_009854705	XP_009854706	XP_009854708	XP_009854709
Verticillium dahliae	VDAG_08037	VDAG_08038	VDAG_08039	VDAG_08040	VDAG_08041
Sclerotinia sclerotiorum	SS1G_04473	SS1G_04475	SS1G_04476	SS1G_04477	SS1G_04472
Botrytis cinerea	BC1G_00448	BC1G_00447	BC1G_00446	BC1G_00445	BC1G_00451
Arthrobotrys oligospora	XP_011120260	XP_011123749	XP_011119552	XP_011117653	XP_011119554

*, identities are at the amino acid level to the corresponding homologue of *A. fumigatus*.
