# Supplementary material for: Conservative production of galactosaminogalactan in Metarhizium is responsible for appressorium mucilage production and topical infection of insect hosts
Source: PLoS Pathog. 2021 Jun 14;17(6):e1009656. doi: 10.1371/journal.ppat.1009656 (PMC8224951; doi:10.1371/journal.ppat.1009656)
Supplement: S2 Table — (PDF) [file ppat.1009656.s011.pdf]

S2 Table. Estimation and comparison the median lethal time (LT_{50}, hours) between WT and different mutants during topical infection of the wax moth larvae.

Strains	LT_{50}	χ^2*	***P***
WT	84 ± 4.19	–	–
Δ*MrGAG*	108 ± 6.57	7.621	0.006
Δ*MrAgd*	132 ± 7.77	28.528	0
Δ*MrEga*	108 ± 6.08	9.52	0.002
Δ*MrSph*	96 ± 5.83	6.263	0.012
Δ*MrUge*	108 ± 9.68	6.775	0.009
Δ*MrGtb*	108 ± 8.06	6.139	0.013

*, Log-rank test of the survival difference between WT and Δ*MrGAG*.
