# Supplementary material for: Conservative production of galactosaminogalactan in Metarhizium is responsible for appressorium mucilage production and topical infection of insect hosts
Source: PLoS Pathog. 2021 Jun 14;17(6):e1009656. doi: 10.1371/journal.ppat.1009656 (PMC8224951; doi:10.1371/journal.ppat.1009656)
Supplement: S4 Table — (PDF) [file ppat.1009656.s013.pdf]

S4 Table. Verification and comparison of the selected protein expressions detected by PRM and iTRAQ analyses.

Protein name	Protein annotation	PRM Ratio (MrGAG/WT)	iTRAQ ratio (MrGAG/WT)
MAA_09630	glycosyl hydrolase family 27	0.80	0.770
MAA_08563	Aminopeptidase	1.93	1.256
MAA_07696	Carboxypeptidase	0.95	0.846
MAA_05630	Cell wall galactomannoprotein Mp2	1.22	0.905
MAA_07347	Glutathione S-transferase	1.25	1.148
MAA_06565	Glutathione S-transferase/chloride channel	2.18	1.258
MAA_06336	Glycoside hydrolase family 61	1.32	1.247
MAA_01100	Heat shock protein	1.64	1.091
MAA_08921	Lipase 2	1.06	0.911
MAA_06941	MrAgd, Extracellular serine-rich protein	0	0.389
MAA_06934	Peptidase family T4 protein	1.67	1.215
MAA_05820	Peptidase M24, structural domain protein	1.75	1.206
MAA_03262	Peptidase S1/S6, chymotrypsin/Hap	0.86	0.717
MAA_09372	Peptidase S28	1.77	1.248
MAA_08819	Perilipin-like protein, Mpl1	1.78	1.177
MAA_00492	Subtilisin-like protease	1.81	1.105
MAA_09454	Thioredoxin-like fold protein	1.45	1.036
