# Supplementary material for: Conservative production of galactosaminogalactan in Metarhizium is responsible for appressorium mucilage production and topical infection of insect hosts
Source: PLoS Pathog. 2021 Jun 14;17(6):e1009656. doi: 10.1371/journal.ppat.1009656 (PMC8224951; doi:10.1371/journal.ppat.1009656)
Supplement: S5 Table — (PDF) [file ppat.1009656.s014.pdf]

S5 Table. PCR primers used in this study.

Primer	Sequence	RE*	Application
MrSph			
KO UF	AGCTTGATATCGAATTCACGACTGACATCTTTAGCT	EcoR I	Gene deletion
KO UR	ATCATCTTCTGTCGACCATCTACGTGACGGACGAG		
KO DF	GTCACCGAGATCTGATGAGTTGACAATGGCGACGAAC	Sac I	
KO DR	GGAACAAAAGCTGGAGCTCCAGGAATAGGTAGTAGTAC		
TF	TCGTCCGTCACGTAGATG		PCR verification
TR	GTTCGTCGCCATCGTCAAC		
Sph-RE.F	GAGATCTGATGAACTAGTCGTCGTTTGCGAGCCTGCAG		Gene rescue
Sph-RE.R	GAACAAAAGCTGGAGCTCTCAACTTTCCGAGCACTC		
RT-F	TCTTTGAGCAGTCGCATC		RT-PCR
RT-R	GTTAGCTCGGTCCACAC		
MrAgd			
KO UF	CAGCCCGGGGGATCCGACCAGACGAAATCTACAG	BamH I	Gene deletion
KO UR	CAATATCATCTTCTGTCGACGATCCTACCTGGTCTCAGTC		
KO DF	CACCGAGATCTGATGACATATGCAACATTGGAGATG	Spe I	
KO DR	CCGCTCTAGAACTAGTGTGAAGCTTGACAAACGAC		
TF	GTAGTAGTAGCAATGCCTC		PCR verification
TR	CTCGACGGCTATGGCATAC		
Agd-RE.F	GCAGCCCGGGGGATCCCTACAGCTTGACCGGCGT		Gene rescue
Agd-RE.R	GCACGTCGACGGATCCGTTGTCGTTTTGCTAGTC		
RT-F	CGTCGTCATCTCCAATGT		RT-PCR
RT-R	CACGATGGTGCCAAATTC		
MrEga			
KO UF	CTGCAGCCCGGGGGATCCGTGCCATTATTATCATATGC	BamH I	Gene deletion
KO UR	CAATATCATCTTCTGTCGACGAAGTAGCAAATGACGCTG		
KO DF	CACCGAGATCTGATGAGACAACTTTAGCACCGTC	Spe I	
KO DR	CGCTCTAGAACTAGTCATCTTCTTCGACGAGATG		
TF	CACGACGTGGCAAATCATC		PCR verification
TR	GTTTGTGGCTTGTTCCAG		
Ega-RE.F	GCAGCCCGGGGGATCCTCGGTATTGCTTCCGCAAC		Gene rescue
Ega-RE.R	GCACGTCGACGGATCCTCAGCTAAAGATGTCAGTCG		
RT-F	CCGAACCTCCATCTACAAG		RT-PCR
RT-R	GCGCTGAAGTAGCAAATG		
MrUge			
KO UF	CAGCCCGGGGGATCCGTTGACAATGGCGACGAAC	BamH I	Gene deletion
KO UR	ATCATCTTCTGTCGACCATCACGCAGGTGCTCAC		
KO DF	CACCGAGATCTGATGACTTGAAGCTGTTGCTGAG	Spe I	
KO DR	CGCTCTAGAACTAGTGTGTATTGTAGAAACAGAC		
TF	CTTGGATACGAAGTTCCAC		PCR verification
TR	CTCAGCAACAGCTTCAAG		
RT-F	CCTTGTCCCATCCAGAAT		RT-PCR
RT-R	CATACAACGCTTGAGCTTC		
MrGtb			
KO UF	CTGCAGCCCGGGGGATCCGTCATGTTTGAGTCGACCAG	BamH I	Gene deletion
KO UR	ATCATCTTCTGTCGACGACGCCGAAAAAGTACGAG		
KO DF	CACCGAGATCTGATGACTTTGAGTTTGGCACCATG	Spe I	
KO DR	GCTCTAGAACTAGTCAGTACTTTTGCAGCGTC		

TF	CTCGTACTTTTTCGGCGTC	PCR verification
TR	GTCCGTCGACTCAATGTAG	
RT-F	CTCATCCTGTTTGGCATTAC	RT-PCR
RT-R	GCCACGACATCAGAGTATAG	
MrGAG		
KOUF	GATAAGCTTGATATCGAATTCCTGCAAGTCGTAAAAGGCAG	Gene deletion
KOUR	GTTGGCACGTCGACGGATCCCTCGACGGCTATGGCATAC	
KODF	CACGTCGACTAGTTCTAGAGAATGGTATCAACAAGCGAC	
KODR	GGTGGCGGCCGCTCTAGAGTACCGGTCGTATATGATG	
TF	CAAGTAGCTTTGGCGCTTG	PCR verification
TR	CTAGCGCAGAATTTGGTGAG	

*RE, restriction enzyme.
